# Reveromycins A and B from *Streptomyces* sp. 3–10: Antifungal Activity against Plant Pathogenic Fungi *In vitro* and in a Strawberry Food Model System

**DOI:** 10.3389/fmicb.2017.00550

**Published:** 2017-04-03

**Authors:** Ang Lyu, Hao Liu, Hongjie Che, Long Yang, Jing Zhang, Mingde Wu, Weidong Chen, Guoqing Li

**Affiliations:** ^1^State Key Laboratory of Agricultural Microbiology, Key Laboratory of Plant Pathology of Hubei Province, Huazhong Agricultural UniversityWuhan, China; ^2^United States Department of Agriculture, Agricultural Research Service, Washington State UniversityPullman, WA, USA

**Keywords:** *Streptomyces* sp. 3–10, reveromycins, plant pathogenic fungi, strawberry, antifungal activity, biological control

## Abstract

This study was conducted to determine the antifungal activity of the metabolites from *Streptomyces* sp. 3–10, and to purify and identify the metabolites. Meanwhile, the taxonomic status of strain 3–10 was re-evaluated. The cultural filtrates of strain 3–10 in potato dextrose broth were extracted with ethyl acetate. The resulting crude extract at 1 and 5 μg/ml inhibited growth of 22 species in 18 genera of plant pathogenic fungi and Oomycetes, accounting for 92% of the total 24 tested species, suggesting that it has a wide antifungal spectrum. Two compounds were purified from the crude extract and were identified as reveromycins A and B, which demonstrated high antifungal activity against *Botrytis cinerea, Mucor hiemails, Rhizopus stolonifer*, and *Sclerotinia sclerotiorum* under acidic pH conditions. Both the crude extract and reveromycin A from strain 3–10 at 10, 50, and 100 μg/ml showed high efficacy in suppression of strawberry fruit rot caused by the above-mentioned four pathogens. The efficacy was comparable to that of corresponding commercial fungicides (pyrimethanil, captan, dimetachlone) used in management of these pathogens. Morphological, physiological, and phylogenetic characterization showed that strain 3–10 is closely related to *Streptomyces yanglinensis* 1307^T^, representing a novel phylotype in that species. This study reported a new strain with reveromycins-producing capability. The finding is important for further exploitation of reveromycins for agricultural use.

## Introduction

*Streptomyces* species (Order: *Streptomycetales*, family: *Streptomycetaceae*) are Gram-positive, filamentous, and spore-producing actinobacteria with high G + C content in their genomes. They are generally regarded as pharmaceutically important biological resources, because many species of *Streptomyces* can produce various physiologically active metabolites, including antibiotics, antimicrobial enzymes, antioxidants, as well as anti-inflammatory and anti-tumor compounds. It is estimated that up to 80% bioactive metabolites are produced by species of *Streptomyces* in Streptomycetales (Bérdy, 2012).

*Streptomyces* as well as other actinobacteria widely live or dwell in various terrestrial environments, including soil, plant, and air dust (Kettleson et al., 2013; Guo et al., 2015; Supong et al., 2016). While a few species of *Streptomyces* are plant pathogens (e.g., *S. scabies* caused potato scabies), majority of *Streptomyces* species live as saprophytes in soil or live as endophytes in plant tissues. The non-pathogenic *Streptomyces* usually produce beneficial effects on plant growth either through providing plants with nutrients from degradation of complex biological polymers like cellulose and chitin in soil (Brzezinska et al., 2013), or through secretion of plant hormones like indole-3-acetic acid, gibberellic acid, and zeatine (Solans et al., 2011). Meanwhile, many species of *Streptomyces* have been found to be capable of suppressing growth, development, and/or survival of plant pathogens (fungi, bacteria, nematodes, viruses) through diverse mechanisms, including production of antibiotics and antimicrobial enzymes (Anitha and Rabeeth, 2010; Zacky and Ting, 2013; Mander et al., 2016), hyperparasitism (Chen et al., 2016), and induction of plant resistance response (Conn et al., 2008; Lehr et al., 2008; Kurth et al., 2014). Therefore, *Streptomyces* species are important resources of biofungicides or biofertilizers for agricultural use.

Attempts to use *Streptomyces* species to control plant diseases can be traced back to 1927, when Millard and Taylor (1927) found that *Streptomyces praecox* (an obligate saprophyte, formerly known as *Actinomyces praecox*) effectively suppressed potato scabies when it was applied in soil either alone or together with green manures. Streptomycin produced by *S. griseus* was the first antibiotic successfully used for control of *Erwinia amylovora*, the causal agent of pear fire blight (Beer et al., 1984). Since then, extensive studies have been carried out to exploit *Streptomyces* as biofungicides and/or biofertilizers. Many *Streptomyces* species have been successfully developed into commercial biofungicides either based on their live spores such as Mycostop® (Minuto et al., 2006) and Rhizovit® (Berg et al., 2010), or based on their bioactive metabolites such as blasticidin S, kasugamycin, polyoxins, and validamycins (Kim and Hwang, 2007). Due to these successful applications, discovering new *Streptomyces* strains and identifying novel antibiotics from *Streptomyces* species continue to be a hot and attractive research area. Many novel antibiotics such as bafilomycin K produced by *Streptomyces flavotricini* Y12-26 (Zhang et al., 2011), elaiomycins B produced by *Streptomyces* sp. BK190 (Kim et al., 2011) and novonestmycins produced by *S. phytohabitans* (Wan et al., 2015) are being evaluated as new biofungicides.

Reveromycins are the antibiotics first isolated from the cultures of *Streptomyces reveromyceticus* SN-593 in the early 1990s (Osada et al., 1991). Reveromycin A showed inhibitory effect on growth and proliferation of the human pathogen *Candida albicans* and the human cancer cell lines KB and K562 (Osada et al., 1991). Osada et al. (1991) indicated that reveromycin A had antifungal activity against plant pathogenic fungi. However, they did not provide the detailed information about the plant pathogenic fungi inhibited by reveromycin A in that report (Osada et al., 1991). Therefore, whether or not reveromycin A and other reveromycins can be used to control plant fungal diseases remains unknown.

Wan et al. (2008) isolated an antagonistic strain (F-1) of *S. platensis* from a healthy leaf of rice (*Oryza sativa* L.). They found that *S. platensis* F-1 is an effective biocontrol agent for suppression of many plant pathogenic fungi, including *Botrytis cinerea, Rhizoctonia solani*, and *Sclerotinia sclerotiorum*, the causal agents of tomato gray mold, rice sheath blight, and Sclerotinia stem rot of oilseed rape, respectively (Wan et al., 2008). In order to improve the antifungal activity of *S. platensis* F-1, Che (2011) conducted a study to mutate the wild type strain F-1 by combined treatments of the spores of strain F-1 with UV-C and LiCl. A putative mutant named strain 3–10 was found to have enhanced antifungal activity by approximately 100 times compared to that of the original strain F-1 (Che, 2011). However, the chemical identity of the antifungal metabolites produced by the putative mutant strain 3–10 remains unknown. Moreover, strain 3–10 differed greatly from the original strain F-1 in colony morphology on potato dextrose agar, implying that it may not be derived from *S. platensis* F-1 (Che, 2011). Therefore, the taxonomic status of strain 3–10 needs to be clarified.

The objectives of this study are: (i) to determine the antifungal spectrum of the crude extract from the cultures of *Streptomyces* sp. 3–10; (ii) to purify and identify the antifungal metabolites produced by *Streptomyces* sp. 3–10; (iii) to evaluate the efficacy of the metabolites from strain 3–10 in suppression of strawberry fruit rot caused by *B. cinerea, Mucor hiemails, Rhizopus stolonifer*, and *S. sclerotiorum*; and (iv) to clarify the taxonomic status of *Streptomyces* sp. 3–10.

## Materials and methods

### Microbial species and cultural media

Five species of bacteria and 24 species of fungi and Oomycetes were used in this study and their origins were listed in Table S1. Among these microbial species, *Streptomyces* sp. 3–10 was used for production of antifungal metabolites. It was isolated from a culture containing spores of *S. platensis* F-1 jointly treated by UV-irradiation and LiCl (Che, 2011). *Aspergillus niger* A-1 (a soil saprophyte) was used as indicator in bioassays to detect the antifungal activity of the metabolites of strain 3–10 (Shakeel et al., 2016). The remaining two species of Oomycetes, four species of bacteria, and 21 species of fungi were used to test the antimicrobial spectrum of the metabolites of *Streptomyces* sp. 3–10.

A total of 22 cultural media (BM, CDM, GA, GS-1, ISM, ISP-1 to ISP7, ISP-9, KB, NA, NB, OCD, PDA, PDB, SDM, TDM, and TYM) were used in this study. The full names of these media and their ingredient compositions were listed in Table S2.

### Preparation of the crude extract

Spore suspension (1 × 10^8^ spores/ml) of *Streptomyces* sp. 3–10 was inoculated in 250-ml Erlenmeyer flasks each containing 100 ml potato dextrose broth (PDB), 1 ml spore suspension per flask. The flasks were incubated on a rotary shaker (150 rpm) at 28°C for 3 days for production of antifungal metabolites. The culture supernatant was collected by centrifugation at 6,000 × g for 10 min, and then extracted with ethyl acetate. The ethyl acetate phase was then dried by vacuum using R-250 Rotavapor® (BUCHI Corporation, New Castle, USA) at 37°C and, a kind of brownish colloidal substance was obtained. It was treated as the crude extract of the antifungal metabolites of *Streptomyces* sp. 3–10.

### Determination of the antimicrobial spectrum of the crude extract

Inhibition of fungi and Oomycetes was done using the PDA-amendment method. The crude extract (CE) dissolved in methanol at 12.5 mg/ml (w/v) was used as stock solution, which was then amended to PDA in Petri dishes (9 cm diameter, 20 ml per dish) to the final concentrations of 1 or 5 μg/ml. PDA amended with methanol alone (0.2%, v/v) was treated as control. Mycelial agar plugs (5 mm diameter) were removed from the margin area of the actively-growing colonies of the target organisms, and inoculated on the PDA amended with either the crude extract or methanol alone, one mycelial plug per dish, and three dishes (as three replicates) for each treatment. The resulting cultures were incubated at 20°C, 25°C, or 28°C for 1–7 days depending on the mycelial growth rates of the target organisms (Table 1). Diameter of the colony in each dish was measured. Inhibition of mycelial growth (IMG) was calculated using the formula: IMG (%) = (D_CK_ − D_CE_)/D_CK_ × 100, where the D_CK_ and D_CE_ represent the colony diameters in the treatments of control (CK) and CE, respectively.

**Table 1:** **Antifungal spectrum of the metabolites of *Streptomyces* sp. 3–10 (PDA, pH 5.3)**.

**Fungus/omycetes**	**% Inhibition of growth (Means** ±**S.D.)**		**Cultural conditions (temperature, duration)**
	**1 μg/ml**	**5 μg/ml**	
**OOMYCOTA**			
*Pythium apanidermatum*	40.5 ± 2.8	73.2 ± 0.9	20°C at 4 dpi^a^
*Pythium ultimum*	20.1 ± 1.7	48.2 ± 0.5	20°C at 1 dpi
**ZYGOMYCOTA**			
*Mucor hiemails*	80.8 ± 2.5	100.0	20°C at 2 dpi
*Rhizopus stolonifer*	91.4 ± 2.8	100.0	20°C at 1 dpi
**ASCOMYCOTA**			
*Amphobotrys ricini*	69.3 ± 0.9	83.7 ± 1.4	20°C at 7 dpi
*Alteraria alternata*	38.7 ± 0.6	74.5 ± 7.5	25°C at 5 dpi
*Aspergillus flavus*	84.1 ± 2.7	95.2 ± 0.6	28°C at 3 dpi
*Aspergillus niger*	76.6 ± 1.3	97.5 ± 0.7	28°C at 3 dpi
*Aspergillus parasiticus*	23.6 ± 7.2	45.8 ± 1.4	28°C at 3 dpi
*Bipolaris maydis*	27.7 ± 8.1	52.7 ± 0.6	28°C at 5 dpi
*Botrytis cinerea*	86.0 ± 0.2	97.0 ± 0.6	20°C at 3 dpi
*Colletotrichum siamense*	79.8 ± 0.6	87.1 ± 1.5	28°C at 3 dpi
*Curvularia lunata*	17.2 ± 2.7	85.3 ± 0.7	25°C at 3 dpi
*Drechslera graminea*	82.7 ± 0.9	100.0	25°C at 3 dpi
*Fusarium graminearum*	0.2 ± 0.1	1.5 ± 0.1	25°C at 3 dpi
*Fusarium moniliforme*	28.9 ± 5.3	57.2 ± 4.7	25°C at 3 dpi
*Fusarium oxysporum*	83.6 ± 1.0	100.0	20°C at 5 dpi
*Monilia fructigena*	75.5 ± 3.3	94.3 ± 0.8	25°C at 3 dpi
*Pestalotia theae*	57.5 ± 5.0	87.7 ± 2.9	25°C at 3 dpi
*Pyricularia oryzae*	3.6 ± 2.5	9.4 ± 5.6	28°C at 3 dpi
*Sclerotinia minor*	92.5 ± 0.3	100.0	20°C at 2 dpi
*Sclerotinia sclerotiorum*	97.2 ± 0.3	100.0	20°C at 2 dpi
**BASIDIOMYCOTA**			
*Rhizoctonia solani*	16.6 ± 4.5	84.7 ± 2.4	28°C at 3 dpi
*Sclerotium rolfsii*	57.1 ± 1.8	91.8 ± 0.4	28°C at 3 dpi

a *dpi, days post-incubation*.

Inhibition of the bacteria by the CE of *Streptomyces* sp. 3–10 was done using the agar diffusion method (Bonev et al., 2008). The four investigated bacteria (Table S1) were separately shake-incubated at 28°C (150 rpm) in nutrient broth for 48 h. Then, aliquots (100 μl) of the liquid culture of each bacterium were evenly spread on the nutrient agar in Petri dishes (9 cm diameter, 20 ml medium per dish). Sterilized stainless-steel Oxford cups (10 × 6 × 8 mm, height × inner diameter × outer diameter) were placed in the dishes, three cups per dish and three dishes for each bacterium. The three concentrations (10, 50, and 100 μg/ml) of the CE of *Streptomyces* sp. 3–10 were pipetted into the three Oxford cups in each dish, respectively, 200 μl per cup. For the control treatment, three Oxford cups in a bacteria-inoculated Petri dish were loaded with 1% methanol (v/v), 200 μl per cup. The cultures were incubated at 28°C for 48 h and formation of the clear zones around the cups was used as an indicator of antibacterial activity.

### Suppression of fungal spore germination by the crude extract

*Rhizopus stolonifer* and *Botrytis cinerea* were incubated on PDA at 20°C for 3 and 7 days, respectively. Spores of *R*. *stolonifer* (sporangiospores) and *B. cinerea* (conidia) were harvested from the respective cultures by washing with sterile distilled water and the spore suspensions (1 × 10^6^ spores/ml) were then prepared. Aliquots (100 μl) of the spore suspensions were pipetted to and evenly spread on the D-glucose agar (GA) medium amended with the crude extract of *Streptomyces* sp. 3–10 at 0 (control), 1, 5, 10, 50, and 100 μg/ml, six dishes (as six replicates) for each treatment. The cultures were incubated at 20°C in the dark for 6, 9, and 12 h. At each time point, the number of germinated spores among randomly-selected at least 100 spores in each culture were counted under a compound light microscope. Additionally, length of the germ tubes of randomly-selected 20 germinated spores was measured in each replicate culture at 12 h post-incubation.

### Isolation of the antifungal metabolites from the crude extract

The crude extract (10 g) of *Streptomyces* sp. 3–10 was dissolved in 15 ml methanol and the solution was mixed with 15 g 60-mesh silica-gel granules (Qingdao Haiyang Chemical Co., Ltd., China). The mixture was then loaded as the top layer in a chromatography column (4.5 × 80 cm, inner diameter × length) containing 400 g silica gel (200-mesh, Qingdao Haiyang Chemical Co., Ltd.). The column was eluded with gradient chloroform/methanol solutions (from 99/1 to 0/100, v/v). The resulting fractions were individually assayed for antifungal activity against *A. niger* using the Oxford cup-agar diffusion method described by Shakeel et al. (2016). The fractions that had antifungal activity were combined, concentrated, and loaded in a Sephadex LH-20 chromatography column (GE Healthcare), which was eluded with methanol to remove impurities. The fractions that had antifungal activity were again combined, dried, and subjected to semi-preparative high performance liquid chromatography (HPLC). Finally, two pure compounds, designated as Compound Nos. 1 and 2, were obtained.

### Identification of the purified compounds

Electrospray ionization mass spectrometric (ESI-MS) analysis and nuclear magnetic resonance spectroscopy (NMR) were used to determine the chemical structure of the two pure compounds from *Streptomyces* sp. 3–10. The ESI-MS analysis was done on a Waters ACQUITY UPLC *H-Class* system coupled to the XEVO TQ-S tandem quadrupole (Waters Cooperation, Milford, MA, USA). The compounds were separately dissolved in methanol to the final concentration of 1 μg/ml. An aliquot (1 μl) of each solution was injected into the instrument. The operating parameters were set as: capillary voltage at 1.0 kV, cone voltage at 30 kV, Z-spray source temperature at 100°C, desolvation temperature (N_2_) at 400°C, desolvation gas flow at 800 L/h, mass range of *m/z* from 50 to 1,200. The mass spectra were collected both in the positive mode and in the negative mode, and compared with those in Chapman Combined Chemical Dictionary on CD-ROM version 6.1 (Chapman and Hall, 2003) for determination of the chemical identity of the compounds. Moreover, synthetic reveromycin A (purity > 98%) purchased from Abcam Trading (Shanghai) Co. Ltd. (Shanghai, China) was used as a reference chemical in the UV-Vis spectrum analysis on Waters ACQUITY UPLC *H-Class* system.

In the NMR analysis, the two pure compounds were dissolved in methanol-d_4_ (CD_3_OD, Sigma-Aldrich®), and were then determined in a 400 MR DD2 spectrometer (Agilent Technologies, USA) for spectra of ^1^H-NMR and ^13^C-NMR. Tetramethylsilane (TMS) was used as the internal standard in the NMR analysis.

### Determination of the content of reveromycin a in the crude extract

Synthetic reveromycin A (purity > 98%) purchased from Abcam Trading (Shanghai) Co. Ltd. was used as standard in quantitative determination of the content of reveromycin A in the CE from strain 3–10. Five solutions of the synthetic reveromycin A with the concentrations of 0.08, 0.16, 0.31, 0.63 and 1.25 μg/ml in methanol were prepared. An aliquot (1 μl) of each solution of the synthetic reveromycin A or the solution of the CE of strain 3–10 at 3.125 μg/ml was injected into Waters ACQUITY UPLC *H-Class* system. The peak area for reveromycin A in each solution sample was measured. A standard curve was plotted based on the peak areas for the solutions of the synthetic reveromycin A and the concentrations of those solutions. The content of reveromycin A in the CE of strain 3–10 was then calculated based on that standard curve. The quantitative determination was repeated for three times.

### Antifungal activity of the purified compounds

The purified compounds were separately dissolved in methanol to 10 mg/ml (w/v) as stock solutions, which were used in the following two trials, a mycelial growth trial and a spore germination trial. In the mycelial growth trial, the stock solution of each compound was amended in PDA, which was adjusted to pH 4.5, 5.5, or 7.0 with 1 mol/L HCl or 1 mol/L NaOH. The final concentrations in PDA ranged from 0.3125 to 50.0 μg/ml for Compound No. 1, and from 0.3125 to 100 μg/ml for Compound No. 2 (Table S3). Meanwhile, methanol solution (0.2%, v/v) was added to PDA at pH 4.5, 5.5, or 7.0 as controls. Mycelial agar plugs of *B. cinerea, M*. *hiemails, R*. *stolonifer*, and *S*. *sclerotiorum* (5 mm diameter) were individually inoculated on PDA, three dishes for each treatment (fungus × compound × concentration × pH). The cultures were incubated at 20°C in the dark for 1 day for *R. stolonifer*, and for 3 days for the other three fungi. The colony diameter was measured and the percentage value of inhibition of mycelial growth (IMG) under each pH was calculated using the formula mentioned above. EC_50_-values (effective concentrations that gave 50% inhibition) for each compound under each pH were estimated based on the IMG data and the concentrations of that compound applied in PDA (Van Ewijk and Hoekstra, 1993).

In the spore germination trial, the stock solution of each compound was amended in GA with Ph-values of 4.5, 5.5, or 7.0. The final concentrations in GA ranged from 0.0625 to 50.0 μg/ml for Compound No. 1, and from 0.3125 to 100 μg/ml for Compound No. 2 (Table S3). Meanwhile, methanol solution (0.2%, v/v) was added to GA with pH 4.5, 5.5, and 7.0 as controls. Aliquots (100 μl) of the spore suspension (1 × 10^6^ spores/ml) of *B. cinerea, M*. *hiemails*, or *R*. *stolonifer* were pipetted on GA alone or on GA amended with a purified compound, three dishes for each treatment (fungus × compound × concentration × pH). The cultures were incubated at 20°C for 12 h. Spore germination in each culture was observed under light microscope. Percent inhibition of spore germination (ISG) by each compound under each pH was calculated using the formula: ISG (%) = (P_CK_–P_C_)/P_CK_ × 100, where the P_CK_ and P_C_ represent the percentages of germinated spores in the treatments of control (CK) and a pure compound, respectively. Finally, EC_50_-values for each compound under each pH were estimated based on the ISG data and the concentrations of that compound applied in GA (Van Ewijk and Hoekstra, 1993).

### Control of strawberry fruit rot by antifungal metabolites from strain 3–10

Mature strawberries (*Fragaria* × *ananassa* cultivar “Jing Yu”) of similar size (3.0–3.5 × 2.0–2.5 cm, length × width, 16–18 g per berry) were collected from strawberry plants grown in a plastic tunnel. They were surface sterilized in 70% ethanol (v/v) for 1 min, followed by washing in sterile distilled water and blotting on sterilized paper towels to remove the excess water on the fruit surface. Finally, they were placed in glass Petri dishes (9 cm diameter), six berries per dish. For each pathogen, there were 10 treatments, including one negative control treatment with 1% methanol (v/v), three CE treatments with the concentrations of 10, 50, and 100 μg/ml (pHs at 5.4, 4.3, and 4.2, respectively), three treatments of reveromycin A (from strain 3–10) with the concentrations at 10, 50, and 100 μg/ml (pH at 4.5), and three fungicide treatments (positive controls) with the concentrations of an appropriate fungicide (either captan, pyrimethanil, or dimetachlone) at 10, 50, and 100 μg a.i./ml. Captan (3a, 4, 7, 7a-tetrahydro-2-[(trichloromethyl) thio]-1 *H-*isoindole-1,3(2*H*)-dione) was used as the positive control for the pathogens *M*. *hiemails* and *R*. *stolonifer*, whereas pyrimethanil (2-anilino-4,6-dimethylpyrimidine) and dimetachlone [N-(3,5-dichlorophenyl) succinimide] were used as positive controls for the pathogens *B. cinerea* and *S. sclerotiorum*, respectively. Both captan (50% wettable powder) and pyrimethanil (80% water dispersible granule) were purchased from Hebei Guan Long Agrichemical Co. Ltd. (Hengshui, China). Dimetachlone (40% wettable powder) was purchased from Zhejiang SPACE Agrichemical Co. Ltd. (Wenzhou, China).

The six strawberries in a Petri dish were immerged for 1 min either in the 1% methanol solution, or in a solution containing either the CE, reveromycin A or a fungicide. Then, they were maintained in a laminar flow hood for 15–30 min, re-placed in the dish, and inoculated with one of the four pathogens. *B*. *cinerera, M*. *hiemails*, and *R*. *stolonifer* were spray-inoculated with the spore suspensions (1 × 10^6^ spores/ml) amended with 0.5% D-glucose (w/v), approximately 10 ml spore suspension on the six strawberries in a Petri dish. *S*. *sclerotiorum* was inoculated with mycelial agar plugs (5 mm diameter), one mycelial agar plug on each berry. The dishes with the treated strawberries were placed in plastic boxes (80 × 60 × 50 cm, length × width × height), which were individually covered with a transparent plastic film (0.1 mm thick, Gold Mine Plastic Industry Ltd., Jiangmen, China) to maintain the high humidity condition. The boxes were placed in a growth room at 20°C under the light regime of 12-h light/12-h dark. After incubation for 3 and 5 days for the inoculation treatments with *R*. *stolonifer* and *M*. *hiemails*, respectively, and for 7 days for the inoculation treatments with *B*. *cinerea*, and *S*. *sclerotiorum*, disease severity on the strawberries was individually rated using a numeric scale from 0 (completely healthy) to 8 (completely rotten) according to the description by Huang et al. (2011). Disease severity index (DSI) was then calculated using the following formula:

$$\text{DSI}\;=100\;\times \sum_{i\;=\;0}^{n}\left({\text{S}}_{n}\times \;n\right)/8\;\times \;\sum_{i\;=\;0}^{n}\left({\text{S}}_{n}\right)\;$$

Where *n* represents the rating scale (0–8) and *S*_*n*_ represents the number of strawberries corresponding to the rating scale *n*. This experiment was repeated two more times.

### Determination of phytotoxicity

The strawberry seedlings (*Fragaria* × *ananassa* cultivar “Jing Yu”) were trans-planted in a plastic tunnel (Length × Width × Height, 29 × 6.5 × 2 m) in September 28 of 2016. The plants were carefully managed (watering, weeding) as required. The toxicity experiment was performed from December 11 of 2016 to December 25 of 2016, when most strawberry plants became bloomed. There were four treatments in this experiment: (i) control (CK); (ii), (iii), and (iv) the crude extract of strain 3–10 (CE) at 10, 50, and 100 mg/ml, respectively. Twenty strawberry plants for each treatment were randomly selected in the field and the leaves on each selected plants were individually immerged for 1 min either in 1% methanol (v/v) (CK) or in each aqueous solution of CE. At 0, 1, 3, 7, and 14 days post treatment (dpt), the treated plants were observed for the toxicosis symptoms on the leaves (yellowing, necrosis and malformation on leaves and flowers). Three plants for each treatment were randomly selected and the three leaves on each treated plant were detached as a leaf sample, one leaf from each plant. The leaves were immediately taken to laboratory for determination of the chlorophyll content (a, b and total). For chlorophyll extraction, the three-leaf sample for each treatment was homogenized in 95% ethanol. The homogenate was centrifuged at 10,000 × g and the supernatant was transferred out for determination of the absorbance values in DU730 Beckman spectrophotometer at 663 and 645 nm, respectively (Rout et al., 1998). The resulting absorbance values A_663_ and A_645_ were used to calculate the content of chlorophyll a (Chl a), chlorophyll b (Chl b) and the total chlorophyll (Chl T) using the following formula:

Chl a (mg/g. F.W.) = [12.72 × A_663_ − 2.59 × A_645_] × V/W
Chl b (mg/g. F.W.) = [22.88 × A_645_ − 4.67 × A_663_] × V/W
Chl T (mg/g. F.W.) = [20.29 × A_645_ + 8.05 × A_663_] × V/W

where V represents volume of 95% ethanol used for chlorophyll extraction in homogenization; W represents weight of the leaf sample; F. W. represent fresh weight of the leaf sample.

Additionally, at 0 or 14 dpt, five other plants for each treatment were randomly selected, uprooted and again taken to laboratory. They were washed under the running tap water and dried in an oven at 50°C. The upper part of that plant (the roots were trimmed) was then weighed.

### Morphological and physiological characterization

*Streptomyces* sp. 3–10 was streak-inoculated on various agar media (Tables S2, S5). The cultures were incubated at 28°C in the dark for 14 days for observation of the colony morphology (shape, size, color of the substrate mycelium and the aerial mycelium, soluble pigments in media). For morphological observation of the substrate mycelium, a sterilized glass slide (7.5 × 2.5 × 0.1 cm, length × width × thickness) was placed on a PDA culture of strain 3–10 at 28°C for 14 days. Then, the slide was removed from the culture, and the hyphae on the slide were stained with 1% methyl green and observed under a compound light microscope (Ruan and Huang, 2011). For observation of the spore morphology, a sterilized cellophane film was placed on PDA. Strain 3–10 was inoculated on the cellophane film and the culture was incubated at 28°C for 14 days. Then, the cellophane film was removed, and cut to small pieces (approximately 3 × 3 mm, length × width). The resulting film pieces with the colonies of strain 3–10 were immediately fixed in the glutaraldehyde fixative, followed by dehydration with ethanol, drying in a Critical Point Dryer (Model: 13200E-AB, SPI SUPPLIES, West Chester, PA, USA), and gold-coating in a sputter coater (Model: JFC-1600, NTC, Tokyo, Japan) using the conventional procedures. Finally, the specimens on the film pieces were observed under a scanning electron microscope (Model: JSM-6390/LV, NTC, Tokyo, Japan). To characterize physiological features, strain 3–10 was inoculated on specific media (Tables S2, S6, S7) using related procedures described by Ruan and Huang (2011).

### Phylogenetic analysis

*Streptomyces* sp. 3–10 was shake-incubated at 28°C for 2 days in the liquid ISP-2 medium (Table S2). The mycelia in the cultures were harvested by centrifugation at 7,000 × g for 5 min. Genomic DNA (gDNA) was extracted from the mycelium using the reagents in the Wizard® Genomic DNA Purification Kit (Promega, Madison, WI, USA). It was used as template in PCR for amplification of the 16S rDNA sequence using the universal primers 27f (5′-AGAGTTTGATCCTGGCTCAG-3′) and 1492r (5′-TACGGCTACCTTGACGACTT-3′; Zhang et al., 2016). The 25-μl PCR reaction system contained 1 μl of gDNA (approximately 50 ng), 2.5 U *Taq* DNA polymerase (TaKaRa Biotechnol. Co., Ltd., Dalian, China), 2.5 μl 10 × PCR buffer, 1 μl of each primer (20 μmol) and 0.5 μl of dNTPs mixture (10 mmol/L). The PCR was performed in a S1000^TM^ thermal cycler (Bio-Rad, USA) with the following thermal program: initial denaturation at 95°C for 5 min, followed by 35 cycles (95°C for 30 s, 56°C for 30 s, 72°C for 1.5 min), and final extension at 72°C for 5 min. The resulting PCR product was purified from the agarose gel after electrophoresis and cloned into the pMD18-T vector (TaKaRa Biotechnol Co. Ltd., Dalian, China), which was subsequently transformed into *E. coli* DH5α. A positive *E. coli* clone with the correct DNA insert size was sequenced at Sangon Biotechnol. Co. Ltd. (Shanghai, China). The sequence was submitted to GenBank at NCBI and was assigned with the accession number KX811537.

For phylogenetic analysis, a dataset was established based on the 16S rDNA sequences of *Streptomyces* sp. 3–10, 39 other taxa of *Streptomyces* species, and strain DSM44928 of *Catenulispora acidiphila* (Table S4). They were aligned using the Clustal W program in the MEGA 7.0 software and phylogenetic analysis was done based on the alignment using the maximum likelihood (ML) methods. All the nucleotides in the DNA sequences were treated as un-ordered and un-weighted, and the gaps were treated as the missing data. The bootstrap consensus trees were inferred from 1,000 replicates.

### Statistical analysis

Data on spore germination rate, length of germ tubes, disease incidence and disease severity index in related experiments were separately analyzed for ANOVA (analysis of variance) using the PROC ANOVA procedure (SAS Institute, Cary, NC, USA, version 8.0, 1999). Treatment means in each experiment were separated using Least significance Different (LSD) test at α = 0.05. Before ANOVA, the percentage data on spore germination rate and disease incidence were transformed to numerical data by multiplication with 100. After analysis, the data were back-transformed to the percentage values. Data on content of chlorophyll a, chlorophyll b, the total chlorophyll and dry weight of strawberry plant between the treatments of control and the crude extract of *Streptomyces* sp. 3–10 was compared at α = 0.05 using the PROC TTEST in the SAS software.

## Results

### Antifungal spectrum of the crude extract

The crude extract from *Streptomyces* sp. 3–10 showed a wide antifungal spectrum (Table 1). It inhibited mycelial growth of two species of *Pythium* and 20 species of fungi. The percentages of inhibition of mycelial growth varied greatly among the target species, ranging from 16.6 to 97.2% at 1 μg/ml, and from 45.8 to 100% at 5 μg/ml. Among the 20 fungal species, *M. hiemails, B. cinerea, R. stolonifer*, and *S. sclerotiorum* are the causal agents of strawberry fruit rot. They were inhibited by 80.8, 86.0, 91.4, and 97.2%, respectively, at 1 μg/ml, and by the rates higher than 97% at 5 μg/ml (Table 1). In contrast, the crude extract weakly inhibited (<10%) mycelial growth of *Fusarium graminearum* (the causal agent of wheat head blight) and *Pyricularia oryzae* (the causal agent of rice blast) at two concentrations (Table 1).

Results from the antibacterial experiment showed that the crude extract of *Streptomyces* sp. 3–10 had no antibacterial activity (Figure S1). It failed even at 100 μg/ml to inhibit growth and proliferation of all the four investigated bacteria, including *Acidovorax citrulli* (the causal agent of watermelon bacterial fruit blotch), *Curtobacterium flaccumfaciens* pv. *flaccumfaciens* (the causal agent of bacterial wilt of beans), and *Erwinia carotovora* (the causal agent of potato soft rot), and *Bacillus subtilis* (a saprophyte).

### Suppression of fungal spore germination by the crude extract

The crude extract effectively inhibited spore germination of *B. cinerea* and *R. stolonifer* in GA at 20°C (Table 2). For *B. cinerea*, the inhibitory efficacy depended greatly on concentration of the crude extract. In the treatments of the crude extract at 1 and 5 μg/ml, the conidia germinated by 93–99% at 6–12 h post-incubation (hpi), not significantly different (*P* > 0.05) from the germination rate of 99% in the control treatment. The average values of germ-tube length reached 146.6 and 106.5 μm for the two concentrations of the crude extract, respectively. In the treatments of the crude extract at 10, 50, and 100 μg/ml, the percentages of germinated conidia at 12 hpi were reduced by 12, 90, and 99%, respectively, and the average germ tube length was reduced by 51, 89, and 92%, respectively, compared to the control treatment (Table 2). For *R. stolonifer*, while the control treatment had percentages of germinated sporangiospores of 0, 8.3 and 63.1% at 6, 9, and 12 hpi, respectively, and the average germ-tube length reached 67.4 μm at 12 hpi, the treatments of the crude extract at 1, 5, 10, 50, and 100 μg/ml completely inhibited sporangiospore germination at 6, 9, and 12 hpi, just as the fungicide treatment with captan at 100 μg a.i./ml (Table 2).

**Table 2:** **Effect of the crude extract (CE) of the antifungal substances produced by *Streptomyces* sp. 3–10 on spore germination and germ tube extension of conidia of *B. cinerea* and sporangiospores of *R. stolonifer* (20°C, GA)**.

**Fungus**	**Treatment^a^**	**Spore germination (%)**			**Length of germ tubes (μm, 12 hpi)**
		**6 hpi^b^**	**9 hpi**	**12 hpi**	
*B. cinerea*	Control	95.1 a^c^	96.6 a	98.7 a	147.3 a
	CE 1 μg/ml	94.5 a	95.7 a	98.8 a	146.6 a
	CE 5 μg/ml	93.5 a	96.5 a	98.2 a	106.5 b
	CE 10 μg/ml	39.3 b	81.5 b	86.7 b	72.3 c
	CE 50 μg/ml	7.5 c	10.9 c	9.9 c	17.0 d
	CE 100 μg/ml	0.8 d	0.9 d	1.0 d	11.4 d
	Pyrimethanil 100 μg a.i./ml	0.8 d	0.9 d	1.1 d	9.3 d
	LSD (0.05)	2.2	1.7	2.4	7.9
*R. stolonifer*	Control	0.0 a	8.3 a	63.1 a	67.4 a
	CE 1 μg/ml	0.0 a	0.0 b	0.0 b	0.0 b
	CE 5 μg/ml	0.0 a	0.0 b	0.0 b	0.0 b
	CE 10 μg/ml	0.0 a	0.0 b	0.0 b	0.0 b
	CE 50 μg/ml	0.0 a	0.0 b	0.0 b	0.0 b
	CE 100 μg/ml	0.0 a	0.0 b	0.0 b	0.0 b
	Captan 100 μg a.i./ml	0.0 a	0.0 b	0.0 b	0.0 b
	LSD (0.05)	0.0	0.3	4.6	3.2

a *pH-values in GA alone, and in GA amended with the crude extract of Streptomyces sp. 3–10 at 1, 5, 10, 50, and 100 μg/ml were 5.8, 5.9, 5.8, 5.4, 4.8, and 4.6, respectively*.

b *hpi, hours post-incubation*.

c *Means within the same column for each fungus followed by the same letter are not significantly different (P > 0.05) according to least significance test*.

### Chemical identity of the purified compounds

**Figure 1:**
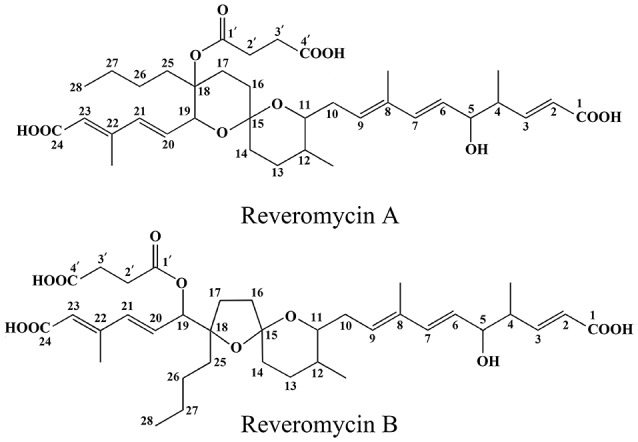
**Chemical structures of reveromycins A and B produced by *Streptomyces* sp. 3–10**.

Two compounds (Nos. 1 and 2) were purified from the crude extract. Compound No. 1 is a white amorphous powder. ESI±MS (100 kV) of this compound showed a molecular ion peak at *m/z* 683 (M+Na)^+^, 659 (M−H)^−^ and HR-ESI (positive) MS at *m/z* 683.3394 (M+Na)^+^ (calc., 683.3047). Based on these data, the molecular formula of this compound was inferred to be C_36_H_52_O_11_. The UV-Vis spectrum in methanol gave the UV maximum absorbance at 237.9 nm (Figure S2). The ^1^H-NMR spectrum (CD_3_OD, 400 MHz; Figure S3) showed five methyl protons at δ_H_ 2.26 (3H, s), 1.75 (3H, s), 1.08 (3H, d, *J* = 6.8 Hz), 0.85 (3H, t, *J* = 6.9 Hz), and 0.79 (3H, d, *J* = 5.6 Hz), eight olefinic protons at δ_H_ 6.97 (1H, dd, *J* = 15.7, 7.7 Hz), 6.44 (2H, m), 6.25 (1H, d, *J* = 15.6 Hz), 5.88 (1H, s), 5.81 (1H, d, *J* = 15.7 Hz), 5.59 (1H, t, *J* = 7.0 Hz), and 5.53 (1H, dd, *J* = 15.6, 7.3 Hz), and three oxymethine protons at δ_H_ 4.62 (1H, d, *J* = 7.3 Hz), 4.07 (1H, m), and 3.45 (1H, m; Table 3). The ^13^C NMR spectrum (CD_3_OD, 100 MHz; Figure S4) showed 36 signals, including five methyl groups at δ_C_ 14.6, 15.3, 13.0, 18.0, and 14.2, 10 methylene carbons at δ_C_ 32.7, 28.7, 36.9, 35.2, 25.4, 34.8, 23.8, 23.4, 31.2, and 29.9, eight olefinic carbons at δ_C_ 122.7, 152.6, 127.9, 137.8, 129.5, 134.1, 139.3, and 121.9, two methine carbons at δ_C_ 44.1 and 36.3, two olefinic quaternary carbons at δ_C_ 135.5 and 152.2, four oxymethine carbons at δ_C_ 76.9, 76.3, 84.2, and 79.7, three carboxyl carbonyl carbons at δ_C_ 170.3, 170.4, and 176.2, one ester carbonyl carbon at δ_C_ 173.4, and one quaternary spiroketal carbon at δ_C_ 97.0 (Table 3). Compound No. 1 was identified as reveromycin A (Figure 1) by comparing the data on the spectra of ^1^H NMR, ^13^C NMR, MS and UV-Vis of this compound with the related spectra of reveromycin A reported by Fremlin et al. (2011). The average content of reveromycin A in the crude extract of strain 3–10 was 37.7% (Figure S5).

**Table 3:** **^1^H and ^13^C NMR spectra of reveromycins A and B from *Streptomyces* sp. 3–10**.

**Reveromycin A**			**Reveromycin B**		
**Position**	**δ_H_ [m, *J*(Hz)]**	**δ_C_**	**Position**	**δ_H_ [m, *J*(Hz)]**	**δ_C_**
1		170.3	1		170.4
2	5.81, d (15.7)	122.7	2	5.79, d (15.6)	121.2
3	6.97, dd (15.7, 7.7)	152.6	3	6.99, dd (15.8, 7.5)	152.9
4	2.52, m	44.1	4	2.51, m	44.0
5	4.07, m	76.9	5	4.08, dd (7.4, 5.4)	77.1
6	5.53, dd (15.6, 7.3)	127.9	6	5.47, dd (15.7, 7.6)	127.2
7	6.25, d (15.6)	137.8	7	6.39, d (15.7)	138.6
8		135.5	8		135.2
9	5.59, t (7.0)	129.5	9	5.77, m	130.9
10	2.40, m	32.7	10	2.57, m	32.6
	2.33, m			2.17, m	
11	3.45, m	76.3	11	3.45, m	78.5
12	1.39, m	36.3	12	1.38, m	35.7
13	1.46, m	28.7	13	1.61, m	30.3
14	1.72, m	36.9		1.51, m	
	1.46, m		14	1.72, m	35.3
15		97.0	15		108.6
16	1.84, m	35.2	16	1.99, m	39.8
	1.60, m			1.80, m	
17	2.31, m	25.4	17	1.99, m	32.9
	2.02, td (13.6, 4.0)			1.84, m	
18		84.2	18		88.8
19	4.62, d (7.3)	79.7	19	5.57, d (3.7)	80.4
20	6.44, m	134.1	20	6.24, dd (16.0, 3.8)	132.6
21	6.44, m	139.3	21	6.28, d (16.1)	136.1
22		152.2	22		152.5
23	5.88, s	121.9	23	5.78, s	122.5
24		170.4	24		170.2
25	1.84, m	34.8	25	1.59, m	35.6
	1.68, m			1.47, m	
26	1.25, m	23.8	26	1.31, m	26.6
	1.23, m				
27	1.27, m	23.4	27	1.31, m	24.4
	1.22, m				
28	0.85, t (6.9)	14.6	28	0.92, t (6.9)	14.5
4-Me	1.08, d (6.8)	15.3	4-Me	1.01, d (6.9)	15.1
8-Me	1.75, s	13.0	8-Me	1.74, s	12.7
12-Me	0.79, t (5.6)	18.0	12-Me	0.89, d (6.5)	18.2
22-Me	2.26, s	14.2	22-Me	2.24, s	14.0
1′		173.4	1′		173.1
2′	2.59, m	31.2	2′	2.60, m	30.4
3′	2.59, m	29.9	3′	2.52, m	29.8
4′		176.2	4′		175.9

Compound No. 2 was also a white amorphous powder. ESI±MS (100 kV) of this compound had a molecular ion peak at *m/z* 683 (M+Na)^+^, 659 (M−H)^−^, HR-ESI (positive) MS at *m/z* 683.3041 (M+Na)^+^ (calc., 683.3047). Based on these data, the molecular formula of this compound was inferred to be C_36_H_52_O_11_. The UV spectrum in methanol gave a UV maximum absorbance at 238.9 nm (Figure S6). The ^1^H-NMR spectrum (CD_3_OD, 400 MHz; Figure S7) showed five methyl protons at δ_H_ 2.24 (3H, s), 1.74 (3H, s), 1.01 (3H, d, *J* = 6.9 Hz), 0.92 (3H, t, *J* = 6.9 Hz) and 0.89 (3H, d, *J* = 6.5 Hz), eight olefinic protons at δ_H_ 6.99 (1H, dd, *J* = 15.8, 7.5 Hz), 6.39 (1H, d, *J* = 15.7 Hz), 6.28 (1H, d, *J* = 16.1 Hz), 6.24 (1H, dd, *J* = 16.0, 3.8 Hz), 5.79 (1H, d, *J* = 15.6 Hz), 5.78, (1H, s), 5.77 (1H, m), 5.47 (1H, dd, *J* = 15.7, 7.6 Hz), and three oxymethine protons at δ_H_ 5.57 (1H, d, *J* = 3.7 Hz), 4.08 (1H, dd, *J* = 7.4, 5.4 Hz), 3.45, (1H, m; Table 3). The ^13^C NMR spectrum (CD_3_OD, 100 MHz; Figure S8) also showed 36 signals, including five methyl groups at δ_C_ 14.5, 15.1, 12.7, 18.2, and 14.0, 10 methylene carbons at δ_C_ 32.6, 30.3, 35.3, 39.8, 32.9, 35.6, 26.6, 24.4, 30.4, and 29.8, eight olefinic carbons at δ_C_ 121.2, 152.9, 127.2, 138.6, 130.9, 132.6, 136.1, and 122.5, two methine carbons at δ_C_ 44.0 and 35.7, two olefinic quaternary carbons at δ_C_ 135.2 and 152.5, four oxymethine carbons at δ_C_ 77.1, 78.5, 88.8, and 80.4, three carboxyl carbonyl carbons at δ_C_ 170.4, 170.2, and 175.9, one ester carbonyl carbon at δ_C_ 173.1 and one quaternary spiroketal carbon at δ_C_ 108.6 (Table 3). Compound No. 2 was identified as reveromycin B (Figure 1) by comparing the data on the spectra of ^1^H NMR, ^13^C NMR, MS, and UV-Vis of this compound with related spectra of reveromycin B reported by Fremlin et al. (2011).

### Antifungal activity of the purified compounds

Reveromycins A and B from *Streptomyces* sp. 3–10 effectively suppressed mycelial growth of *B*. *cinerea, M*. *hiemails, R*. *stolonifer*, and *S*. *sclerotiorum*, and spore germination of *B*. *cinerea, M*. *hiemails*, and *R*. *stolonifer* (Table 4). The suppressive efficacy was greatly affected by ambient pH. For reveromycin A, the EC_50_-values ranged from 0.10 to 0.88 μg/ml at pH 4.5, from 0.42 to 1.76 μg/ml at pH 5.5, and from 14.04 to 53.35 μg/ml at pH 7.0. For reveromycin B, the EC_50_-values ranged from 1.15 to 5.49 μg/ml at pH 4.5, from 6.12 to 35.46 μg/ml at pH 5.5, and >100 μg/ml at pH 7.0.

**Table 4:** **50% effective concentrations (EC_50_) of reveromycins A and B produced by *Streptomyces* sp. 3–10 against *B. cinerea, M. hiemails, R. stolonifer*, and *S. sclerotiorum***.

**Fungus**	**Reveromycin A (Means** ±**S.D**., μ**g/ml)**			**Reveromycin B (Means** ±**S.D**., μ**g/ml)**		
	**pH 4.5**	**pH 5.5**	**pH 7.0**	**pH 4.5**	**pH 5.5**	**pH 7.0**
**EC_50_ FOR MYCELIAL GROWTH^a^**						
*B. cinerea*	0.88 ± 0.01	1.76 ± 0.07	53.35 ± 1.01	5.37 ± 0.11	30.60 ± 0.87	>100.00
*M. hiemails*	0.74 ± 0.02	1.49 ± 0.05	21.31 ± 0.32	4.07 ± 0.18	22.12 ± 0.11	>100.00
*R. stolonifer*	0.67 ± 0.03	1.45 ± 0.08	21.45 ± 3.62	4.48 ± 0.04	24.61 ± 0.35	>100.00
*S. sclerotiorum*	0.65 ± 0.02	1.27 ± 0.04	34.03 ± 0.44	5.49 ± 0.13	35.46 ± 3.92	>100.00
**EC_50_ FOR SPORE GERMINATION^b^**						
*B. cinerea*	0.57 ± 0.01	1.45 ± 0.03	49.33 ± 2.11	4.01 ± 0.09	23.62 ± 0.18	>100.00
*M. hiemails*	0.11 ± 0.01	0.45 ± 0.01	15.17 ± 0.56	1.59 ± 0.03	8.03 ± 0.21	>100.00
*R. stolonifer*	0.10 ± 0.01	0.42 ± 0.01	14.04 ± 0.61	1.15 ± 0.01	6.12 ± 0.11	>100.00

a *The cultures on reveromycin A- or reveromycin B-amended PDA were incubated at 20°C for 24 h for R. stolonifer, whereas for 72 h for B. cinerea, M. hiemails and S. sclerotiorum*.

b *The cultures with the conidia of B. cinerea, and sporangiospores of M. hiemails and R. stolonifer on GA were incubated at 20°C for 12 h*.

### Control efficacy against strawberry fruit rot

**Figure 2:**
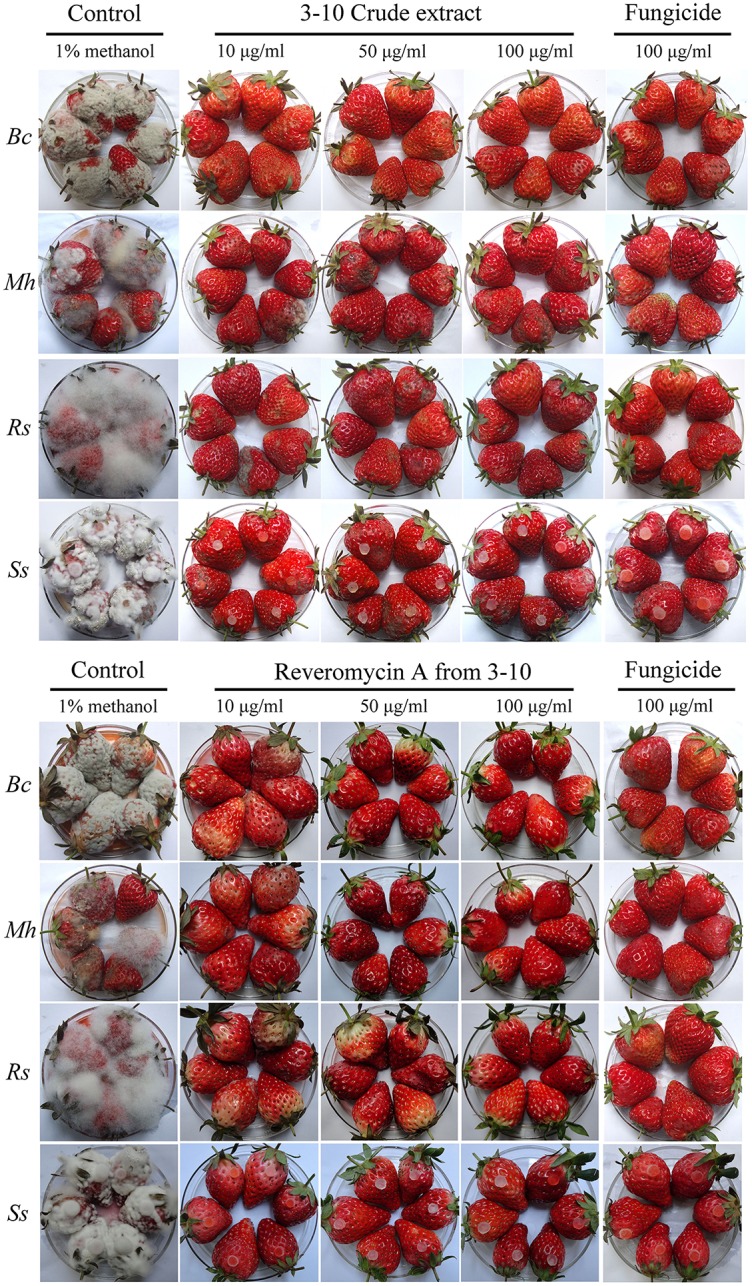
**Efficacy of the crude extract, reveromycin A from *Streptomyces* sp. 3–10 and fungicides in suppression of strawberry fruit rot caused by *Botrytis cinerea* (*Bc*), *Mucor hiemails* (*Mh*), *Rhizopus stolonifer* (*Rs*), and *Sclerotinia sclerotiorum* (*Ss*)**. *B. cinerea, M. hiemails*, and *R. stolonifer* were inoculated with spores. *S. sclerotiorum* was inoculated with mycelial agar plugs. The strawberries were maintained at 20°C under humid conditions for 3 days for *R. stolonifer*, 5 days for *M. hiemails*, and 7 days for *B. cinerea* and *S. sclerotiorum*.

At 3–7 days post inoculation (20°C), the strawberries in the control treatment were severely diseased (Figure 2, Table 5). The percentages of diseased strawberries reached 100, 88.9, 100, and 94.4% for the control inoculations with *B*. *cinerea, M*. *hiemails, R*. *stolonifer*, and *S*. *sclerotiorum*, respectively. The disease severity index values reached 78.5, 62.5, 88.1, and 82.6 in these treatments, respectively. In contrast, most strawberries in the treatments of the crude extract and reveromycin A from strain 3–10 at 10, 50, and 100 μg/ml, as well as in the treatments of the fungicides at 10, 50, and 100 μg a.i./ml appeared healthy (Figure 2, Table 5). The percentages of diseased strawberries were lower than 40% and the disease severity index values were lower than 35 in these treatments. Reveromycin A at 50 and 100 μg/ml completely suppressed strawberry fruit rot caused by all the four fungi. Statistical analysis indicated that for each pathogen, the treatments of the CE, reveromycin A and fungicide differed significantly (*P* < 0.05) from the control treatment both in disease incidence and in disease severity index. Under the same concentration, the crude extract and reveromycin A from strain 3–10 did not significantly (*P* > 0.05) differ from the corresponding fungicide.

**Table 5:** **Efficacy of the crude extract (CE) and reveromycin A (RA) from *Streptomyces* sp. 3–10 in suppression of strawberry fruit rot caused by *B. cinerea, M. hiemails, R. stolonifer*, and *S. sclerotiorum* in comparison with respective fungicides**.

**Treatment ^a^**	***B. cinerea***		***M. hiemails***		***R. stolonifer***		***S. sclerotiorum***	
	**DI^b^**	**DSI^b^**	**DI**	**DSI**	**DI**	**DSI**	**DI**	**DSI**
Control	100.0 a^c^	78.5 a	88.9 a	62.5 a	100.0 a	88.1a	94.4 a	82.6 a
CE 10 μg/ml	22.2 b	4.9 b	11.1 c	8.3 cd	27.7 b	7.6 c	5.6 b	1.4 b
CE 50 μg/ml	11.1 bc	4.2 b	5.6 c	2.1 de	11.1 c	2.8 d	0.0 b	0.0 b
CE 100 μg/ml	0.0 c	0.0 c	0.0 c	0.0 e	0.0 c	0.0 d	0.0 b	0.0 b
RA 10 μg/ml	22.2 b	4.2 b	11.1 c	2.1 e	11.1 c	2.7 d	0.0 b	0.0 b
RA 50 μg/ml	0.0 c	0.0 c	0.0 c	0.0 e	0.0 c	0.0 d	0.0 b	0.0 b
RA 100 μg/ml	0.0 c	0.0 c	0.0 c	0.0 e	0.0 c	0.0 d	0.0 b	0.0 b
FU 10 μg a.i./ml	11.1 bc	3.5 bc	27.7 b	34.7 b	38.8 b	22.8 b	5.6 b	2.1 b
FU 50 μg a.i./ml	5.6 c	2.1 bc	11.1 c	10.4 c	11.1 c	3.5 cd	0.0 b	0.0 b
FU 100 μg a.i./ml	0.0 c	0.0 c	0.0 c	0.0 e	0.0 c	0.0 d	0.0 b	0.0 b
LSD (0.05)	11.6	3.9	12.7	6.3	11.6	4.6	9.0	2.4

a *Fungicides (FU): Pyrimethanil for B. cinerea, captan for M. hiemails and R. stolonifer, and dimetachlone for S. sclerotiorum*.

b *DI, Disease incidence (%); DSI, Disease severity index (0–100)*.

c *Means within the same column followed by the same letter are not significantly different (P > 0.05) according to least significance test*.

### Phytotoxicity

The treatments of the strawberry leaves with the crude extract of *Streptomyces* sp. 3–10 at 10, 50 and 100 μg/ml did not produce any visible toxic symptoms (yellowing, necrosis and malformation) on leaves and flowers (Figure S9). They grew normally and did not significantly differ (*P* > 0.05) from the control treatment (water) both in leaf chlorophyll content and in dry weight of the upper part of strawberry plants.

### Taxonomic identity of *Streptomyces* sp. 3–10

*Streptomyces* sp. 3–10 grew and sporulated on nine agar media (BM, GS-1, ISP-1, ISP-2, ISP-3, ISP-5, ISP-6, ISP-7, PDA) after incubation at 28°C for 14 days with formation of whitish to grayish colonies. They hardly grew on the ISP-4 medium (Figure S10, Table S5). In the PDA cultures, the single colonies of *Streptomyces* sp. 3–10 were averagely sized by 1.6 ± 0.3 mm in diameter, dome-shaped in the colony center, and the saw tooth-shaped at the colony margin (Figure S11). The substrate mycelium was pale yellowish to orange in color and the aerial mycelium was whitish to grayish in color (Figure S11). The spore chains were rectiflexible in shape with the spores being short rod in shape, 0.9 × 0.6 μm (length × width) in size, and smooth surfaced (Figure S11). The cultural and morphological characteristics of *Streptomyces* sp. 3–10 matched the description for *Streptomyces yanglinensis* 1307^T^ (Xu et al., 2006).

Results of the physiological determination showed that strain 3–10 was similar to *S. yanglinensis* 1307^T^ in most of the measured features, but differed from *S. yanglinensis* 1307^T^ in utilization of *myo*-inositol, growth response to pH 3.5, degradation of Tween 80 and sensitivity to streptomycin sulfate and sulfamethoxazole. Strain 3–10 could utilize *myo*-inositol, whereas strain 1307^T^ could not. Strain 3–10 could grow on ISP-3 at pH 3.5, whereas strain 1307^T^ could not. Strain 3–10 could not degrade Tween 80, whereas strain 1307^T^ could. Strain 3–10 showed sensitive to streptomycin sulfate (10 μg/ml), but resistant to sulfamethoxazole (25 μg/ml). In contrast, strain 1307^T^ showed resistant to streptomycin sulfate (10 μg/ml), but sensitive to sulfamethoxazole (25 μg/ml; Tables S6, S7).

**Figure 3:**
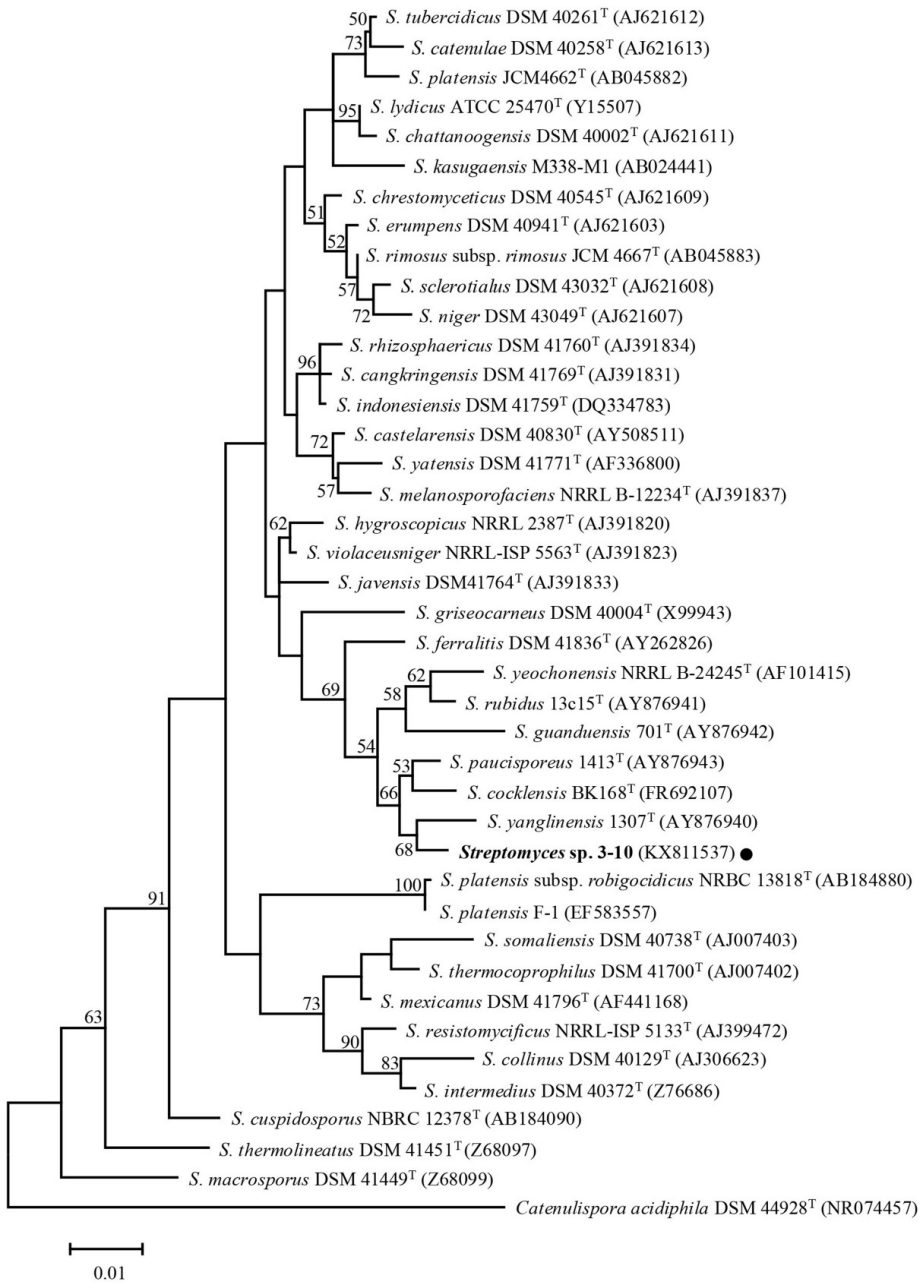
**A maximum-likelihood tree showing the relationship between *Streptomyces* sp. 3–10 and 39 other taxa of *Streptomyces***. The phylogenetic tree was inferred based on 16S rDNA sequences with *Catenuliospora acidiphila* as the out-group. The bootstrap values (*n* = 1,000) higher than 50% are shown at the internodes in the tree. The NCBI GenBank accession numbers are given in parentheses following the strain names. The scale bar indicates 1% nucleotide substitution per site.

The close relationship between *Streptomyces* sp. 3–10 and *S*. *yanglinensis* 1307^T^ was further confirmed by phylogenetic analysis of the 16S rDNA sequences. *Streptomyces* sp. 3–10 is distantly related to strains F-1, JCM4662^T^ and NRBC13818^T^ of *S. platensis*, but is closely related to *S*. *yanglinensis* 1307^T^ (Figure 3). Therefore, *Streptomyces* sp. 3–10 might represent a novel phylotype of *S. yanglinensis*.

## Discussion

Reveromycins are spiroacetal polyketide compounds produced by *Streptomyces* species (Osada et al., 1991; Koshino et al., 1992; Takahashi et al., 1992a,b; Fremlin et al., 2011). So far, three strains of *Streptomyces* have been reported to produce reveromycins. They are strain SN-593 of *S. reveromyceticus* isolated from a soil sample in Japan (Osada et al., 1991; Koshino et al., 1992; Takahashi et al., 1992a,b), and strains MST-MA568 and MST-RA7781 of *Streptomyces* spp. isolated from a sediment sample and a soil sample, respectively, in Australia (Fremlin et al., 2011). Fremlin et al. (2011) reported that the frequency of reveromycins-producing actinomycete isolates is very low. Two (e.g., MST-MA568 and MST-RA7781) out of 400,000 actinomycete isolates were found to be able to produce reveromycins (Fremlin et al., 2011). The present study found that *Streptomyces* sp. 3–10 can produce reveromycins A and B. This finding enriched the reveromycins-producing *Streptomyces* resource and will be important for further exploitation of the *Streptomyces-*derived reveromycins for pharmaceutical and agricultural use in the future.

Biological activities of reveromycin A have been well elucidated in previous studies (Osada, 2016). It is a multifunctional chemical, owning strong capabilities to inhibit mitogenic activity induced by epidermal growth factor (Osada et al., 1991), to suppress hormone-dependent tumors like ovarian cancer and prostate (Takahashi et al., 1997), to alleviate osteoporosis by inducing apoptosis specifically in osteoclasts (Woo et al., 2006), and to inhibit proliferation of *Candida* species (Takahashi et al., 1992b; Fremlin et al., 2011). Miyamoto et al. (2002) found that the molecular target of reveromycin A in *Saccharomyces cerevisiae* is isoleucyl-transfer RNA (tRNA) synthetase. Although Osada et al. (1991) indicated that reveromycin A has antifungal activity against plant pathogenic fungi with the minimum inhibitory concentration ranging from 16 to 64 μg/ml. However, detailed information about the inhibited plant pathogenic fungi was not mentioned in that report (Osada et al., 1991). Therefore, whether or not reveromycin A and other reveromycin analogs can inhibit plant pathogens like *B. cinerea, M. hiemails, R. stolonifer*, and *S. sclerotiorum*, and Oomycetes such as like species of *Pythium* remains unknown. Fremlin et al. (2011) reported that reveromycins A to M had antifungal activity against *Candida* species. They did not report the antifungal activity of these reveromycins against plant pathogenic fungi.

In this study, we found that the crude extract from *Streptomyces* sp. 3–10 at 5 μg/ml had a wide antifungal spectrum, including many important plant pathogens such as, *B. cinerea, M. hiemails, R. stolonifer*, and *S. sclerotiorum* (Table 1). We also demonstrated that the crude extract and reveromycin A from *Streptomyces* sp. 3–10 at 10, 50, and 100 μg/ml was highly effective in suppression of strawberry fruit rot caused by these pathogens and the efficacy was comparable to that of the corresponding commercial fungicides (Table 5). It is well recognized that *B. cinerea, M. hiemails, R. stolonifer*, and *S. sclerotiorum* are the necrotrophic plant pathogens. They can aggressively infect mature strawberry fruit under cool and humid conditions, thereby causing severe economic losses for strawberry production. Nowadays, control of *B. cinerea* as well as three other fungi depends largely on repeated application of fungicides. In most cases, the fungicide application can suppress infection by these fungi. However, frequent application of the fungicides may cause some undesirable side effects, such as fungicide residues in strawberry fruit, pollution to environment, and development of fungicide-resistant fungal strains. The results about the wide antifungal spectrum and high antifungal activity for the metabolites of *Streptomyces* sp. 3–10 suggest that they have a promising potential to be exploited for control of strawberry fruit rot caused by these four fungi. This study found that the crude extract of *Streptomyces* sp. 3–10 at 10, 50 and 100 μg/ml did not produce any visible toxic symptoms on leaves of strawberry (Figure S9). This result suggests that application of the metabolites of *Streptomyces* sp. 3–10 on strawberry plants at the flowering stage might be a safe measure for control of strawberry fruit rot.

Among the fungi and Oomycetes inhibited by the metabolites from *Streptomyces* sp. 3–10, *Fusarium oxysporum, Pythium apanidermatum, P. ultimum, Rhizoctonia solani, Sclerotium rolfsii, Sclerotinia minor*, and *S. sclerotiorum* are soilborne plant pathogens. Antifungal activity against these organisms suggests that inoculation of *Streptomyces* sp. 3–10 into soil may yield suppressive effect on these organisms. This study found that *Streptomyces* sp. 3–10 can grow at pH 3.5 and 4.5, but failed to grow at pH 7.5 (Table S6). This acidophilic characteristic of *Streptomyces* sp. 3–10 may help it to adapt to the acidic soil conditions in south of China. Moreover, this study found that the antifungal activity of reveromycins A and B from *Streptomyces* sp. 3–10 is high at pH 4.5 and 5.5, whereas is decreased at pH 7.0 (Table 4), implying that application of *Streptomyces* sp. 3–10 in acidic soil may achieve high antifungal efficacy in suppression of these soil-dwelling fungi.

Previous studies showed that many plant pathogenic fungi such as *B. cinerea* and *S. sclerotiorum* can secrete oxalic acid to facilitate their infection and colonization of plant tissues (Choquer et al., 2007; Williams et al., 2011). Oxalic acid can acidify the surrounding environment. In this study, we found that reveromycins A and B from *Streptomyces* sp. 3–10 showed higher antifungal activity at pH 4.5 than at pH 5.5 and 7.0 (Table 4). Thus, the acidic environment created by oxalic acid produced by *B. cinerea* and *S. sclerotiorum* may enhance the antifungal activity of *Streptomyces* sp. 3–10 against the two pathogens. This may be one of the reasons responsible for the high antifungal activity of reveromycins A and B in inhibition of *B. cinerea* and *S. sclerotiorum*.

*Streptomyces* sp. 3–10 was previously thought to be a mutant *S. platensis* F-1, as it was isolated from a PDA culture of strain F-1 treated with UV-C and LiCl (Che, 2011). Due to the dramatic difference between strains 3–10 and F-1 in colony morphology, this study tried to clarify the taxonomic status of *Streptomyces* sp. 3–10. The results showed that strain 3–10 is closely related to *S. yanglinensis* 1307^T^ (possibly representing a novel phylotype of *S. yanglinensis*), but is distantly related to *S. platensis* F-1 (Figure 3). This result suggests that *Streptomyces* sp. 3–10 is probably a contaminant, rather than a derivative from *S. platensis* F-1.

*Streptomyces yanglinensis* was established by Xu and colleagues in 2006 with strain 1307^T^ as the type strain (Xu et al., 2006). Results of this study showed that strain 3–10 was similar to strain 1307^T^ in the majority of the measured physiological characteristics, but is different from strain 1307^T^ in utilization of *myo*-inositol, in growth response to pH 3.5, in degradation of Tween 80, as well as in sensitivity to streptomycin sulfate and sulfamethoxazole (Tables S6, S7). Therefore, strain 3–10 may represent a physiological type different from strain 1307^T^.

It is quite interesting that among the four reveromycins-producing strains of *Streptomyces* (SN-593, MST-MA568, MST-RA7781, 3–10) so far known, three strains of *Streptomyces* (MST-MA568, MST-RA7781, 3–10) are closely related to *S. yanglinensis* based on phylogeny of 16S rDNA sequences (Figure 3; Fremlin et al., 2011). This result suggests that *S. yanglinensis*-related species may have a conserved pathway for biosynthesis of reveromycins. Several other strains of *S. yanglinensis* from soil samples collected in China (e.g., strains 317, 913 and 1,307) were readily reported (Xu et al., 2006). Whether or not they can produce reveromycins remains unknown and additional studies are necessary to clarify this point.

## Conclusions

This study demonstrated that the crude extract of *Streptomyces* sp. 3–10 could effectively inhibit mycelial growth of 22 species of fungi and spore germination of *B. cinerea, M. hiemails*, and *R. stolonifer*. Two antifungal compounds, reveromycins A and B, were purified from the cultures of *Streptomyces* sp. 3–10. Both the crude extract of strain 3–10 and reveromycin A purified from that strain showed high efficacy in suppression of strawberry fruit rot caused by *B. cinerea, M. hiemails, R. stolonifer*, and *S. sclerotiorum*. The efficacy was comparable to that of corresponding commercial fungicides. The findings of this study are useful for further exploitation of reveromycins to control plant fungal diseases.

## Author contributions

AL, HL, HC, LY designed research; AL and HL performed research and analyzed the spectra of ^1^H NMR and ^13^C NMR; JZ and MW provided new agents and analyzed the data; AL, WC, and GL wrote the paper.

### Conflict of interest statement

The authors declare that the research was conducted in the absence of any commercial or financial relationships that could be construed as a potential conflict of interest.
