# Supplementary material for: Reveromycins A and B from Streptomyces sp. 3–10: Antifungal Activity against Plant Pathogenic Fungi In vitro and in a Strawberry Food Model System
Source: Front Microbiol. 2017 Apr 3;8:550. doi: 10.3389/fmicb.2017.00550 (PMC5376619; doi:10.3389/fmicb.2017.00550)
Supplement: Supplementary file 1 [file Presentation1.pdf]

Supplementary materials

Reveromycins A and B from *Streptomyces* sp. 3-10: Antifungal Activity against Plant Pathogenic Fungi *In vitro* and in a Strawberry Food Model System

Ang Lyu[1], Hao Liu[1], Hongjie Che[1], Long Yang[1], Jing Zhang[1], Mingde Wu, Weidong Chen[2], and Guoqing Li[1]*

[1]State Key Laboratory of Agricultural Microbiology and Key Laboratory of Plant Pathology of Hubei Province, Huazhong Agricultural University, Wuhan 430070, China; and [2]United States Department of Agriculture, Agricultural Research Service, Washington State University, Pullman, WA, USA.

* Correspondence: Dr. Guoqing Li, E-mail: guoqingli@mail.hzau.edu.cn

I. Supplementary Tables

Supplementary Table S1. List of bacteria, fungi and Oomycetes used in this study

Test fungus and isolates	Host and/or origin*
Bacteria	
Acidovorax citrulli Pslbtw36	Watermelon (*Citrullus lanatus*), HZAU
Bacillus subtilis CanL-30	Oilseed rape (*Brassica napus*), HZAU
Curtobacterium flaccumfaciens pv. *flaccumfaciens*	Bean (*Phaseolus vulgaris*), HZAU
Erwinia carotovora	Chinese cabbage (*B. rapa*), HZAU
Streptomyces sp. 3-10	Unknown
Fungi	
Amphobotrys ricini EEA-25	Copperleaf (*Acalypha australis*), HZAU
Alteraria alternata TBS-3	Tobacco (*Nicotiana tabacum*), HZAU
Aspergillus flavus NRRL3357	Peanut (*Arachis hypogaea*) , NRRL
Aspergillus niger A-1	Soil, HZAU
Aspergillus parasiticus MO527	Rice Rice (*Oryza sativa*), CGMCC
Botrytis cinerea RoseBC-3	Rose (*Rosa chinensis*), HZAU
Bipolaris maydis MB-1	Maize (*Zea mays*), HZAU
Curvularia lunata CFW-4	Rice (*Oryza sativa*), HZAU
Colletotrichum siamense Nj-2	Strawberry (*Fragaria×ananassa*) , HAAS
Drechslera graminea BGW014	Barly (*Hordeum vulgare*), HZAU
Fusarium graminearum FHB-11	*Wheat* (*Triticum aestivum*), HZAU
Fusarium moniliforme WF-3	Rice (*Oryza sativa*) , HZAU
Fusarium oxysporum FO-25	Cotton (*Gossypium hirsutum*), HAAS
Monilia fructigena BT15-56	Peach (*Amygdalus persica*), HZAU
Mucor hiemails WHT-1	Tomato (*Lycopersicon esculentum*), HZAU
Pestalotia theae DMZ21	Rice (*Oryza sativa*), HZAU
Pyricularia oryzae PB-1	Rice (*Oryza sativa*), HZAU
Rhizoctonia solani WH-1	Rice (*Oryza sativa*), HZAU
Rhizopus stolonifer WHS-1	Strawberry (*Fragaria×ananassa*), HZAU
Sclerotinia minor Lc-45	Lettuce *(Lactuca sativa)*, HZAU
Sclerotinia sclerotiorum Ss-1	Oilseed rape (*Brassica napus*), HZAU
Sclerotium rolfsii B-1	Plumepoppy (*Macleaya cordata*), HZAU
Oomycetes	
Pythium ultimum WLOW2F	Watermelon (*Citrullus lanatus*),WAAST
Pythium aphanidermatum Py-xg	Watermelon (*Citrullus lanatus*),WAAST

*NRRL = Agricultural Research Service Culture Collection, United States Department of Agriculture; CGMCC = China General Microbiological Culture Collection Center, Beijing, China: HAAS = Hubei Academy of Agricultural Sciences, Wuhan, China; HZAU = Huazhong Agricultural University, Wuhan, China: WAAST = Wuhan Academy of Agricultural Science and Technology, Wuhan, China.

Supplementary Table S2. Media used in this study and their composition

Medium[a]	Composition (in l,000 ml distilled water)	Reference[b]
BM	D-Glucose 10.0 g, yeast extract 1.0 g, enzymatic hydrolysis casein 2.0 g, beef extract 1.0 g, agar 15.0 g, pH 6.5–7.0	Jones (1949)
CDM	KH_2PO_4 0.3 g, K_2HPO_4 1.0 g, $FeSO_4{\cdot}7H_2O$ 0.015 g, $ZnSO_4{\cdot}7H_2O$ 0.002 g, $MgSO_4{\cdot}7H_2O$, 40 ml 5% colloidal chitin, agar 15.0 g, distilled water 960 ml, pH 6.5–7.0	Guo et al. (2015)
GA	D-Glucose 20.0 g, agar 15.0 g	This study
GS-1	Soluble starch 20.0 g, K_2HPO_4 0.5 g, $MgSO_4{\cdot}7H_2O$ 0.5 g, NaCl 0.5 g, KNO_3 1.0 g, $FeSO_4{\cdot}7H_2O$ 0.01g, agar 15.0 g, pH 6.5–7.0	Zhang et al. (2016)
ISM	$K_2HPO_4{\cdot}3H_2O$ 1.0 g, $MgSO_4{\cdot}7H_2O$ 0.5 g, NaCl 0.5 g, agar 15.0 g, pH 6.5–7.0	Juan and Huang (2011)
ISP-1	Yeast extract 3.0 g, tryptone 5.0 g, agar 15.0 g, pH 6.5–7.0	Shirling and Gottlieb (1966)
ISP-2	Glucose 4.0 g, malt extract 10.0 g, yeast extract 4.0 g, agar 15.0 g, pH 6.5–7.0 (liquid ISP-2 without agar).	Shirling and Gottlieb (1966)
ISP-3	Oatmeal 20.0 g, agar 15.0 g, trace element solution (TES) 1.0 ml (TES: $FeSO_4{\cdot}7H_2O$ 0.1 g, $ZnSO_4{\cdot}7H_2O$ 0.1 g, $MnCl_2{\cdot}4H_2O$ 0.1 g, distilled water 100.0 ml, pH 6.5–7.0	Shirling and Gottlieb (1966)
ISP-4	Soluble starch 10.0 g, K_2HPO_4 1.0 g, $MgSO_4{\cdot}7H_2O$ 1.0 g, NaCl 1.0 g, $(NH_4)_2SO_4$ 2.0 g, $CaCO_3$ 2.0 g, agar 15.0 g, TES 1.0 ml (TES: $FeSO_4{\cdot}7H_2O$ 0.1 g， $ZnSO_4{\cdot}7H_2O$ 0.1 g， $MnCl_2{\cdot}4H_2O$ 0.1 g, distilled water 100.0 ml), pH 6.5–7.0	Shirling and Gottlieb (1966)
ISP-5	Glycerol 10.0 g, L-asparagines 1.0 g, TES 1.0 ml, agar 15.0 g, (TES: $FeSO_4{\cdot}7H_2O$ 0.1 g， $ZnSO_4{\cdot}7H_2O$ 0.1 g， $MnCl_2{\cdot}4H_2O$ 0.1 g, distilled water 100.0 ml), pH 6.5–7.0	Shirling and Gottlieb (1966)
ISP-6	Peptone 20.0 g, yeast extract 1.0 g, K_2HPO_4 1.0 g, ferric citrate 0.5 g, agar 15.0 g, pH 6.5–7.0	Shirling and Gottlieb (1966)
ISP-7	L-tyrosine 0.5 g, L-asparagines 1.0 g, glycerol 15.0 g, $MgSO_4{\cdot}7H_2O$ 0.5 g, $FeSO_4{\cdot}7H_2O$ 0.5 g, NaCl 0.5 g, agar 15.0 g, TES 1.0 ml, agar 15.0 g (TES: $FeSO_4{\cdot}7H_2O$ 0.1 g, $ZnSO_4{\cdot}7H_2O$ 0.1 g, $MnCl_2{\cdot}4H_2O$ 0.1 g, distilled water 100.0 ml), pH 6.5–7.0	Shirling and Gottlieb (1966)
ISP-9	$(NH_4)_2SO_4$ 2.64 g, KH_2PO_4 2.38 g, $K_2HPO_4{\cdot}3H_2O$ 5.65 g, $MgSO_4{\cdot}7H_2O$ 1.0 g, agar 15.0 g, TSE 1.0 ml (TES: $GuSO_4{\cdot}5H_2O$ 0.64 g, $FeSO_4{\cdot}7H_2O$ 0.11 g, $MnCl_2{\cdot}4H_2O$ 0.79 g, $ZnSO_4{\cdot}7H_2O$ 0.15 g, distilled water 100.0 ml, pH 6.5–7.0	Shirling and Gottlieb (1966)
KB	Peptone 20.0 g, $K_2HPO_4{\cdot}3H_2O$ 1.5 g, $MgSO_4{\cdot}7H_2O$ 1.5 g, glycerol 15.0 ml, agar 15.0 g	King et al. (1954)
NA	Beef extract 3.0 g, peptone 5.0 g, agar 15.0 g, pH 7.0	Ruan and Huang (2011)
NB	Beef extract 3.0 g, peptone 5.0 g, pH 7.0	Ruan and Huang (2011)

Continued…

OCD	Glucose 10.0 g, yeast extract 1.0 g, casein 1.0 g, beef extract 1.0 g, agar 15.0 g, adenine 0.2%, guanine 0.2%, xanthine 0.3%, pH 6.5–7.0	Ruan and Huang (2011)
PDA	Peeled potato 200.0 g, D-glucose 20.0 g, agar 15.0 g	Ren et al. (2007)
PDB	Peeled potato 200.0 g, D-glucose 20.0 g	Ren et al. (2007)
SDM	Soluble starch 10.0 g, yeast extract 2.0 g, $MgSO_4 \cdot 7H_2O$ 1.0 g, KNO_3 1.0g, $K_2HPO_4 \cdot 3H_2O$ 0.3 g, NaCl 0.5 g, agar 15.0 g, pH 6.5–7.0	Ruan and Huang (2011)
TDM	Peptone 10.0 g, NaCl 5.0 g, $CaCl_2 \cdot 2H_2O$ 0.1 g, agar 15.0 g, 1% Tween 80 (v/v), pH 6.5–7.0	Ruan and Huang (2011)
TYM	L-tyrosine 1.0 g, yeast extracts 1.0 g, NaCl 8.5 g, agar 15.0 g, pH 6.5–7.0	Ruan and Huang (2011)

[a]Abbreviation:

BM = Bennett's medium; CDM = Chitin degradation medium; GA = Glucose agar; GS-1 = Gauserime synthetic agar medium 1; ISM = Inorganic salt medium; ISP-1, 2, 3, 4, 5, 6, 7, 9 = International *Streptomyces* Project media 1, 2, 3, 4, 5, 6, 7, 9, respectively; KB = King's medium B; NA = nutrient agar; NB = Nutrient broth; OCD = Organic compound degradation medium; PDA = Potato dextrose agar; PDB = Potato dextrose broth; SDM = Starch degradation medium, TYM = Tween degradation medium; TEM = Tyrosine yeast extract medium.

[b] References:

1. Guo, X. X., Liu, N., Li, X. M., Ding, Y., Shang, F., Gao, Y.S., Ruan, J.S., Huang, Y. (2015). Red soils harbor diverse culturable actinomycetes that are promising sources of novel secondary metabolites. *Appl. Environ. Microbiol.* 81, 3086–3013.
2. Jones, K .L. (1949). Fresh isolates of actinomycetes in which the presence of sporogenous aerial mycelia is a fluctuating characteristic. *J. Bacteriol.* 57, 141–145.
3. King, E.O., Ward, M.K., and Raney, D.E. (1954) Two simple media for the demonstration of pyocyanin and fluorescein. J. Lab. Clin. Med. 44, 301–307.
4. Ren, L., Li, G. Q., Han, Y.C., Jiang, D. H., Huang, H. C. (2007). Degradation of oxalic acid by *Coniothyrium minitans* and its effects on production and activity of β-1,3-glucanase of this mycoparasite. *Biol. Contr.* 43, 1–11.
5. Ruan, J. S., Huang, Y. (2011). Rapid identification and systematics of actinobacteria. Science Press: Beijing, pp.313–340;
6. Shirling, E.B., and Gottlieb, D. (1966) Methods for characterization of *Streptomyces* specices. *Int. J. Syst. Bacteriol.* 16, 313–340.
7. Zhang, Y.M., Li, H.Y., Hu, C, Sheng, H.F., Zhang, Y., Lin, B.R., and Zhou, G.X. (2016) Ergosterols from the culture broth of marine *Streptomyces anandii* H41-59. *Mar. Drugs* 14, 84.

Supplementary Table S3. Concentrations (unit: μg/ml) of reveromycins A (No.1) and B (No. 2) from *Streptomyces* sp. 3-10 used for determination of 50% effective concentration values (EC_{50}) in this study

**Reveromycin A (No.1)**: mycelial growth on PDA (pH 4.5)					
B. cinerea	5.0	2.5	1.25	0.625	0.3125
S. sclerotiorum	5.0	2.5	1.25	0.625	0.3125
R. stolonifer	5.0	2.5	1.25	0.625	0.3125
M. hiemails	5.0	2.5	1.25	0.625	0.3125
**Reveromycin A (No.1)**: mycelial growth on PDA (pH 5.5)					
B. cinerea	5.0	2.5	1.25	0.625	0.3125
S. sclerotiorum	5.0	2.5	1.25	0.625	0.3125
R. stolonifer	5.0	2.5	1.25	0.625	0.3125
M. hiemails	5.0	2.5	1.25	0.625	0.3125
**Reveromycin A (No.1)**: mycelial growth on PDA (pH 7.0)					
B. cinerea	50.0	25.0	12.5	6.25	3.125
S. sclerotiorum	50.0	25.0	12.5	6.25	3.125
R. stolonifer	50.0	25.0	12.5	6.25	3.125
M. hiemails	50.0	25.0	12.5	6.25	3.125
**Reveromycin A (No.1)**: spore germination on GA (pH 4.5)					
B. cinerea	1.0	0.5	0.25	0.125	0.06250
R. stolonifer	0.5	0.25	0.125	0.0625	0.03125
M. hiemails	0.5	0.25	0.125	0.0625	0.03125
**Reveromycin A (No.1)**: spore germination on GA (pH 5.5)					
B. cinerea	2.0	1.0	0.5	0.25	0.125
R. stolonifer	2.0	1.0	0.5	0.25	0.125
M. hiemails	2.0	1.0	0.5	0.25	0.125
**Reveromycin A (No.1)**: spore germination on GA (pH 7.0)					
B. cinerea	50.0	25.0	12.5	6.25	3.125
R. stolonifer	50.0	25.0	12.5	6.25	3.125
M. hiemails	50.0	25.0	12.5	6.25	3.125

Continued…

**Reveromycin B (No.2)**: mycelial growth on PDA (pH 4.5)					
B. cinerea	5.0	2.5	1.25	0.625	0.3125
S. sclerotiorum	5.0	2.5	1.25	0.625	0.3125
R. stolonifer	5.0	2.5	1.25	0.625	0.3125
M. hiemails	5.0	2.5	1.25	0.625	0.3125
**Reveromycin B (No.2)**: mycelial growth on PDA (pH 5.5)					
B. cinerea	50.0	25.0	12.5	6.25	3.125
S. sclerotiorum	50.0	25.0	12.5	6.25	3.125
R. stolonifer	50.0	25.0	12.5	6.25	3.125
M. hiemails	50.0	25.0	12.5	6.25	3.125
**Reveromycin B (No.2)**: mycelial growth on PDA (pH 7.0)					
B. cinerea	100	50.0	25.0	12.5	6.25
S. sclerotiorum	100	50.0	25.0	12.5	6.25
R. stolonifer	100	50.0	25.0	12.5	6.25
M. hiemails	100	50.0	25.0	12.5	6.25
**Reveromycin B (No.2)**: spore germination on GA (pH 4.5)					
B. cinerea	5.0	2.5	1.25	0.625	0.3125
R. stolonifer	5.0	2.5	1.25	0.625	0.3125
M. hiemails	5.0	2.5	1.25	0.625	0.3125
**Reveromycin B (No.2)**: spore germination on GA (pH 5.5)					
B. cinerea	20.0	10.0	5.0	2.5	1.25
R. stolonifer	20.0	10.0	5.0	2.5	1.25
M. hiemails	20.0	10.0	5.0	2.5	1.25
**Reveromycin B (No.2)**: spore germination on GA (pH 7.0)					
B. cinerea	100	50.0	25.0	12.5	6.25
R. stolonifer	100	50.0	25.0	12.5	6.25
M. hiemails	100	50.0	25.0	12.5	6.25

Supplementary Table S4. Origin and GenBank accession numbers of 16 rDNA sequences of the isolates of *Streptomyces* species and *Catenuliospora acidiphila* DSM44928 used for phylogenetic analysis of this study

Species	Isolate	GenBank Acc. No.	Reference
Catenulispora acidiphila	DSM44928^{T}	NR074457	Guo et al. (2015)
Streptomyces sp.	3-10	KX811537	Xu et al. (2006)
S. cangkringensis	DSM 41769^{T}	AJ391831	Xu et al. (2006)
S. catenulae	DSM 40258^{T}	AJ621613	Xu et al. (2006)
S. castelarensis	DSM 40830^{T}	AY508511	Xu et al. (2006)
S. chattanoogensis	DSM 40002^{T}	AJ621611	Xu et al. (2006)
S. chrestomyceticus	DSM 40545^{T}	AJ621609	Xu et al. (2006)
S. cocklensis	BK168^{T}	FR692107	Guo et al. (2015)
S. collinus	DSM 40129^{T}	AJ306623	Xu et al. (2006)
S. cuspidosporus	NBRC 12378^{T}	AB184090	Xu et al. (2006)
S. erumpens	DSM 40941^{T}	AJ621603	Xu et al. (2006)
S. ferralitis	DSM 41836^{T}	AY262826	Xu et al. (2006)
S. griseocarneus	DSM 40004^{T}	X99943	Xu et al. (2006)
S. guanduensis	701^{T}	AY876942	Xu et al. (2006)
S. hygroscopicus	NRRL 2387^{T}	AJ391820	Xu et al. (2006)
S. indonesiensis	DSM 41759^{T}	DQ334783	Xu et al. (2006)
S. intermedius	DSM 40372^{T}	Z76686	Xu et al. (2006)
S. javensis	DSM41764^{T}	AJ391833	Xu et al. (2006)
S. kasugaensis	M338-M1	AB024441	Xu et al. (2006)
S. lydicus	ATCC 25470^{T}	Y15507	Xu et al. (2006)
S. macrosporus	DSM 41449^{T}	Z68099	Xu et al. (2006)
S. melanosporofaciens	NRRL B-12234^{T}	AJ391837	Xu et al. (2006)
S. mexicanus	DSM 41796^{T}	AF441168	Xu et al. (2006)
S. niger	DSM 43049^{T}	AJ621607	Xu et al. (2006)
S. paucisporeus	1413^{T}	AY876943	Xu et al. (2006)
S. platensis	JCM4662^{T}	AB045882	Xu et al. (2006)
S. platensis	F-1	EF583557	Wan et al. (2006)
S. platensis subsp. *robigocidicus*	NBRC 13818^{T}	AB184880	Unknown
S. resistomycificus	NRRL-ISP 5133^{T}	AJ399472	Xu et al. (2006)
S. rimosus subsp. *rimosus*	JCM 4667^{T}	AB045883	Xu et al. (2006)
S. rhizosphaericus	DSM 41760^{T}	AJ391834	Xu et al. (2006)
S. rubidus	13c15^{T}	AY876941	Xu et al. (2006)
S. somaliensis	DSM 40738^{T}	AJ007403	Xu et al. (2006)
S. sclerotialus	DSM 43032^{T}	AJ621608	Xu et al. (2006)
S. thermocoprophilus	DSM 41700^{T}	AJ007402	Xu et al. (2006)
S. thermolineatus	DSM 41451^{T}	Z68097	Xu et al. (2006)
S. tubercidicus	DSM 40261^{T}	AJ621612	Xu et al. (2006)
S. violaceusniger	NRRL-ISP 5563^{T}	AJ391823	Xu et al. (2006)
S. yanglinensis	1307^{T}	AY876940	Xu et al. (2006)
S. yatensis	DSM 41771^{T}	AF336800	Xu et al. (2006)
S. yeochonensis	NRRL B-24245^{T}	AF101415	Xu et al. (2006)

References

1. Guo, X. X., Liu, N., Li, X. M., Ding, Y., Shang, F., Gao, Y. S., Ruan, J. S., Huang, Y. (2015). Red soils harbor diverse culturable actinomycetes that are promising sources of novel secondary metabolites. *Appl. Environ. Microbiol.* 81, 3086–3103.
2. Wan, M. G., Li, G. Q., Zhang, J. B., Jiang, D. H., Huang, H.C. (2008) Effect of volatile substance of *Streptomyces platensis* F-1 on control of plant fungal diseases. *Biol. Contr.* 46, 552–559.
3. Xu, C. G., Wang, L. M., Cui, Q. F., Huang, Y., Liu, Z. H., Zheng, G. Y., Goodfellow, M. (2006). Neutrotolerant acidophilic *Streptomyces* species isolated from acidic soils in China: *Streptomyces guanduensis* sp. nov., *Streptomyces paucisporeus* sp. nov., *Streptomyces rubidus* sp. nov. and *Streptomyces yanglinensis* sp. nov. *Int. J. Syst. Evol. Microbiol.* 56, 1109–1115.

Supplementary Table S5. Culture characteristics of *Streptomyces* sp. 3-10 on different media (Streaking inoculation with spores, 28°C, 7 days, dark)

Medium	Growth[a]	Substrate mycelia	Aerial mycelia	Sporulation[b]	Soluble pigment
ISP-1	+	Pale yellowish	Pale yellowish	+	not visible
ISP-2	+++	Orange	Grayish	+++	not visible
ISP-3	++	Pale yellowish	Grayish	+++	not visible
ISP-4	-	N/A	N/A	N/A	N/A
ISP-5	+++	Pale yellowish	Grayish	++	not visible
ISP-6	+++	Pale yellowish	Grayish	++	not visible
ISP-7	++	Pale yellowish	Grayish	++	not visible
GS-1	++	Pale yellowish	Grayish	++	not visible
BM	+++	Dark grayish	Grayish	+++	not visible
PDA	+++	Orange	Grayish	+++	not visible

[a] Growth rating: "-" no growth; "+", forming sparse colonies (whitish); "++" forming dense colonies (light grayish); "+++" forming highly-dense colonies (dark grayish).

[b] Sporulation rating: "+", sparse sporulation; "++", moderate sporulation with powdery appearance on colony surface; "+++", prosperous sporulation with heavy powdery appearance on the colony surface.

Supplementary Table S6. Physiological characteristics of *Streptomyces* sp. 3-10 ("+", positive; "-", negative)

Characteristics	*Streptomyces* sp. 3-10	*S. yanglinensis* 1307$^{T\ a}$
Melanin production (28°C, ISP-6, 14 d)	-	-
Growth on sole carbon sources (1%, w/v, 28°C, ISP-9 as the basal medium)		
Adonitol	+	+
L-Arabinose	+	+
D-Cellobiose	+	+
D-Fructose	+	+
D-Galactose	+	+
Glycerol	+	+
D-Glucose	+	+
D-Maltose	+	+
D-Mannose	+	+
D-Mannitol	+	+
D-Melezitose	-	-
myo-Inositol	+	-
D-Inulin	+	+
D-Raffinose	+	+
L-Rhamnose	+	+
D-Salicin	+	+
D-Sorbitol	+	+
L-Sorbose	+	+
D-Surcose	+	+
D-Trehalose	+	+
D-Xylose	+	+
Utilization of carbon sources (0.1%, w/v, 28°C, ISP-9 as the basal medium)		
L-Alanine	-	-
L-Arginine	-	-
DL-Aminobutyric acid	-	-
L-Cysteine	-	-
L-Phenylalanine	-	-
Sodium acetate	-	-
Sodium citrate	-	-
Sodium oxalate	-	-

Continued…

Growth on sole carbon and nitrogen sources (0.1% in ISM, w/v, 28°C)		
L-Alanine	-	-
L-Arginine	-	-
L-Aspartic acid	-	-
L-Glutamic acid	+	+
L- Isoleucine	-	-
L-Phenylalanine	-	-
Growth on ISP-3 (28°C, 7 d) at:		
pH 3.5	+	-
pH 4.5	+	+
pH 7.5	-	-
Growth at 28°C for 7 days on BM containing NaCl at 5% (w/v)	-	-
Growth temperature (ISP-2, 7 d)	20ºC–37ºC (optimal temp. 28ºC)	20ºC–37 ºC
Degradation of (w/v)		
Adenine (0.2 % in BM)	-	-
Casein (1% in BM)	-	-
Chitin (0.2% in CDM)	+	unknown
Guanine (0.2 % in BM)	-	-
Hypoxanthine (0.4% in BM)	+	+
Soluble starch (1.0 % in SDM)	+	+
Tween 80 (1.0 % in TDM)	-	+
Xanthine (0.3% in BM)	-	-
Xylan (0.4% in BM)	+	+
DNA G+C content	73.8 mol%	74.8 mol%

[a] From the reference: Xu, C. G., Wang, L. M., Cui, Q. F., Huang, Y., Liu, Z. H., Zheng, G. Y., Goodfellow, M. (2006). Neutrotolerant acidophilic *Streptomyces* species isolated from acidic soils in China: *Streptomyces guanduensis* sp. nov., *Streptomyces paucisporeus* sp. nov., *Streptomyces rubidus* sp. nov. and *Streptomyces yanglinensis* sp. nov. *Int. J. Syst. Evol. Microbiol.* 56, 1109–1115.

Supplementary Table S7. Sensitivity of *Streptomyces* sp. 3-10 and *Streptomyces yanglinensis* 1307^T to antibiotics ("+", sensitive, "-", tolerant; 28°C, 5 days)

Antibiotics	Concentration	*Streptomyces* sp. 3-10	*S. yanglinensis* 1307^T [a]
Amoxicillin	10 µg/ml	-	-
Ampicillin	10 µg/ml	-	-
Azithromycin	30 µg/ml	-	-
Aztreonam	30 µg/ml	-	-
Carbenicillium	10 µg/ml	-	-
Cephalothin	30 µg/ml	+	+
Ciprofloxacin	5 µg/ml	-	-
Doxycycline hydrochloride	30 µg/ml	+	+
Erythromycin	15 µg/ml	+	+
Josamycin	15 µg/ml	+	+
Kanamycin sulphate	30 µg/ml	+	+
Neomycin sulphate	30 µg/ml	+	+
Ofloxacin	5 µg/ml	-	-
Rifampicin	5 µg/ml	-	-
Streptomycin sulfate	10 µg/ml	+	-
Sulfamethoxazole	25 µg/ml	-	+
Tetracycline hydrochloride	30 µg/ml	-	-
Tobramycin sulphate	10 µg/ml	+	+

[a] From the reference: Xu, C. G., Wang, L. M., Cui, Q. F., Huang, Y., Liu, Z. H., Zheng, G. Y., Goodfellow, M. (2006). Neutrotolerant acidophilic *Streptomyces* species isolated from acidic soils in China: *Streptomyces guanduensis* sp. nov., *Streptomyces paucisporeus* sp. nov., *Streptomyces rubidus* sp. nov. and *Streptomyces yanglinensis* sp. nov. *Int. J. Syst. Evol. Microbiol.* 56, 1109–1115.

II. Supplementary Figures

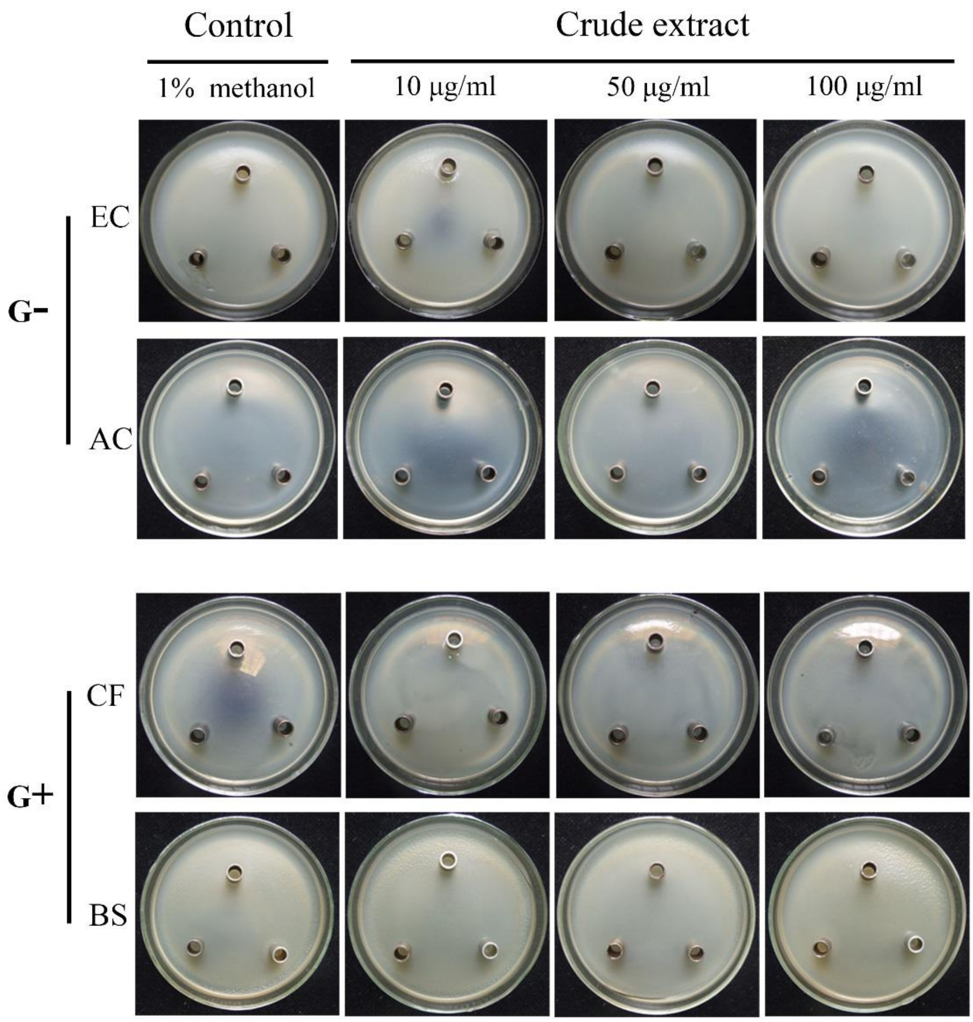

Supplementary Figure S1. Effect of the crude extract of the antifungal metabolites of *Streptomyces* sp. 3-10 on bacterial growth. AC = *Acidovorax citrulli*; BS = *Bacillus subtilis*; CF = *Curtobacterium flaccumfaciens* pv. *flaccumfaciens*; EC = *Erwinia carotovora*. G+ = Gram positive; G- = Gram negative.

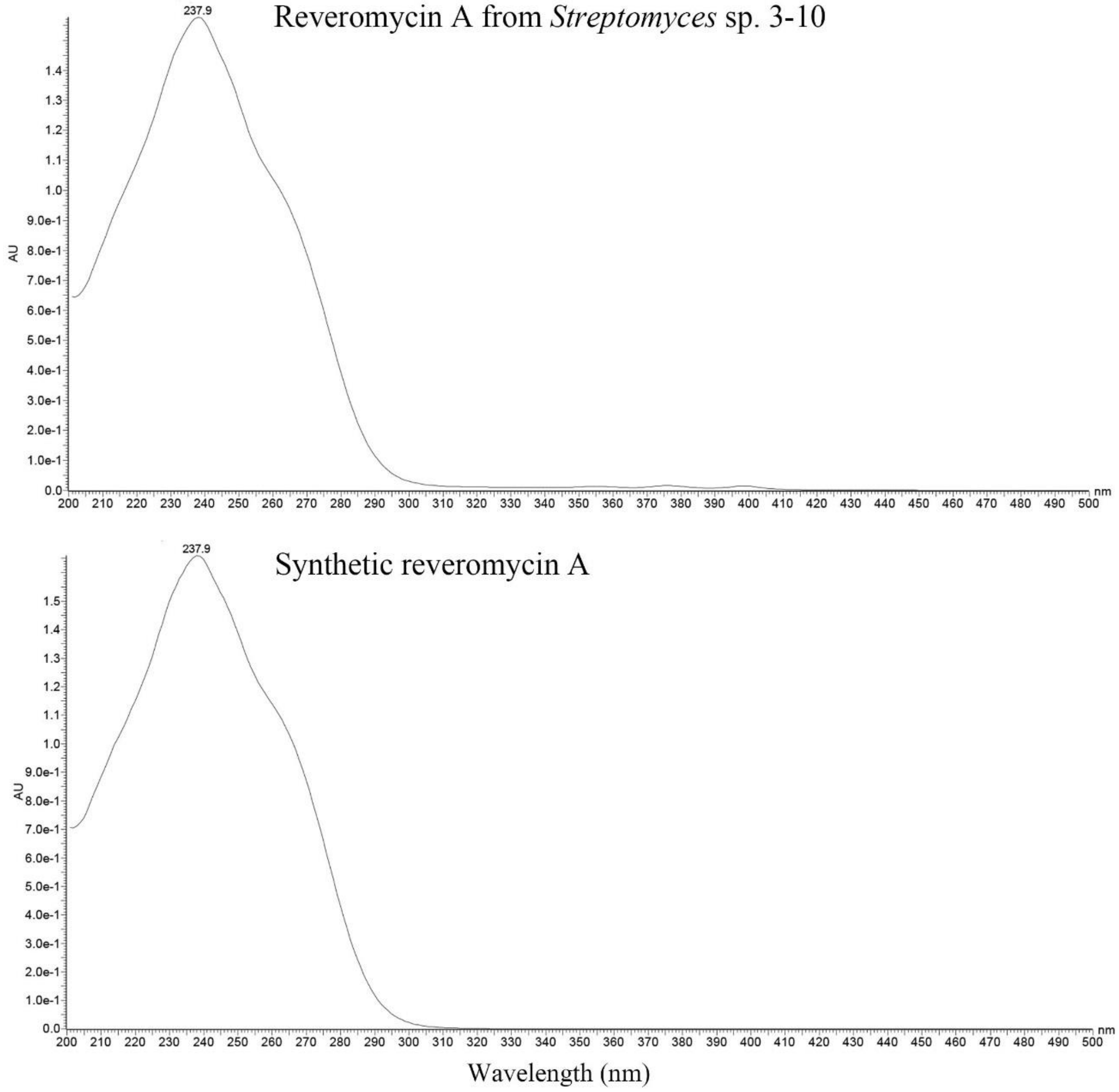

Supplementary Figure S2. UV-Vis spectra of reveromycin A from *Streptomyces* sp. 3-10. Synthetic reveromycin A was used as standard.

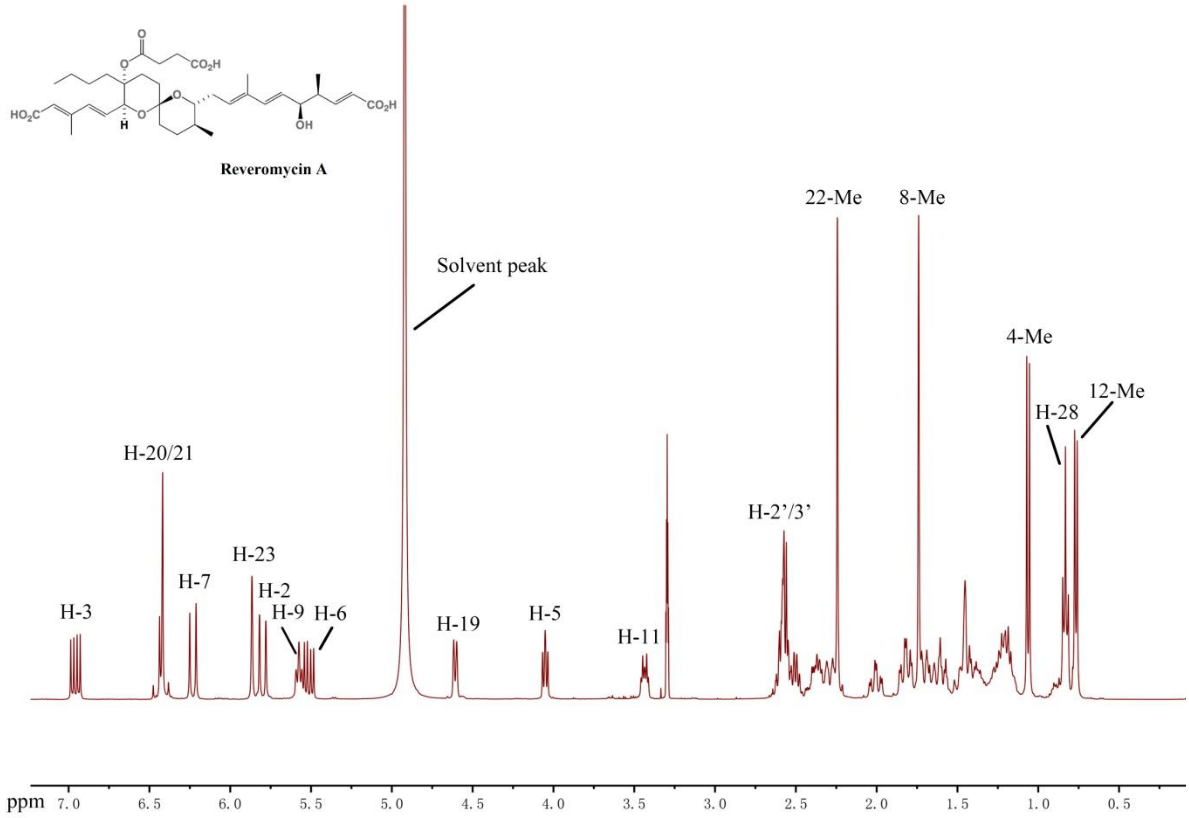

Supplementary Figure S3. ^{1}H NMR (400 MHz, MeOH-d_4, NMR) spectrum for reveromycin A from *Streptomyces* sp. 3-10.

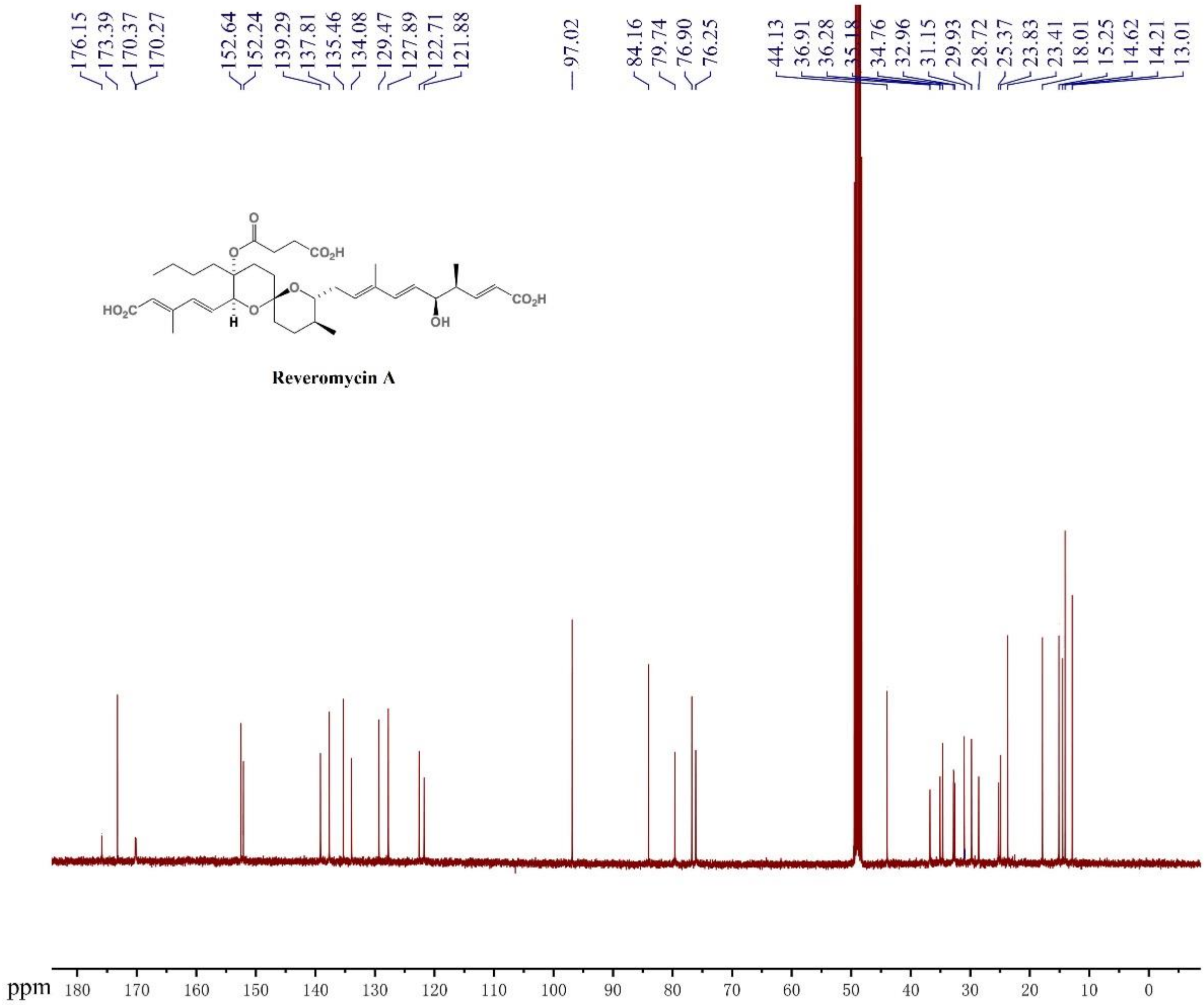

Supplementary Figure S4. ^{13}C NMR (100 MHz, MeOH-d_4, NMR) spectrum for reveromycin A from *Streptomyces* sp. 3-10.

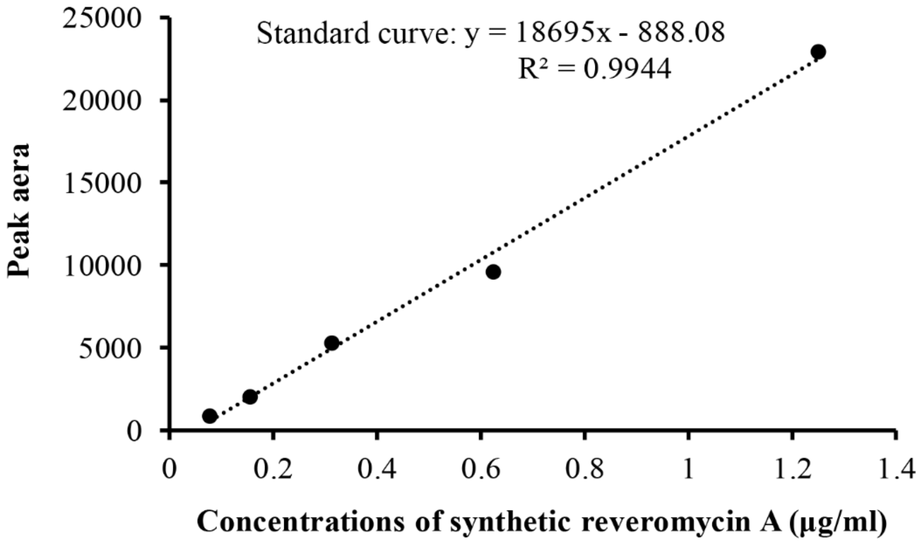

Supplementary Figure S5. Standard curve of synthetic reveromycin A used for measuring reveromycin A content in crude extract from *Streptomyces* sp. 3-10.

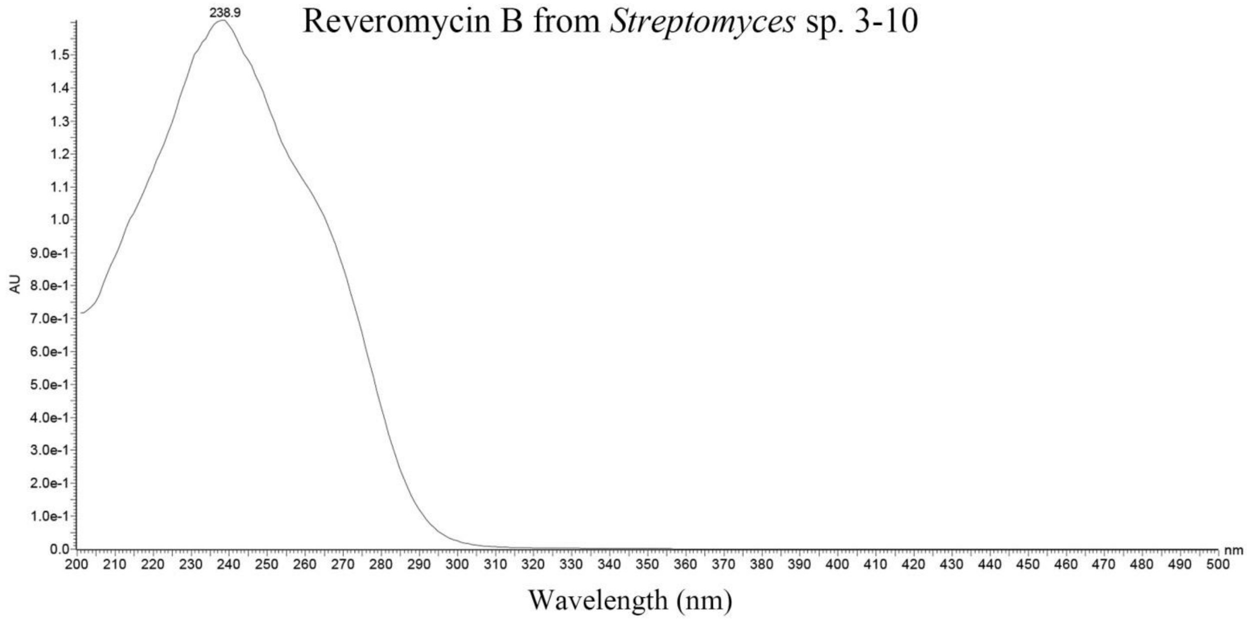

Supplementary Figure S6. UV-Vis spectrum of reveromycin B from *Streptomyces* sp. 3-10.

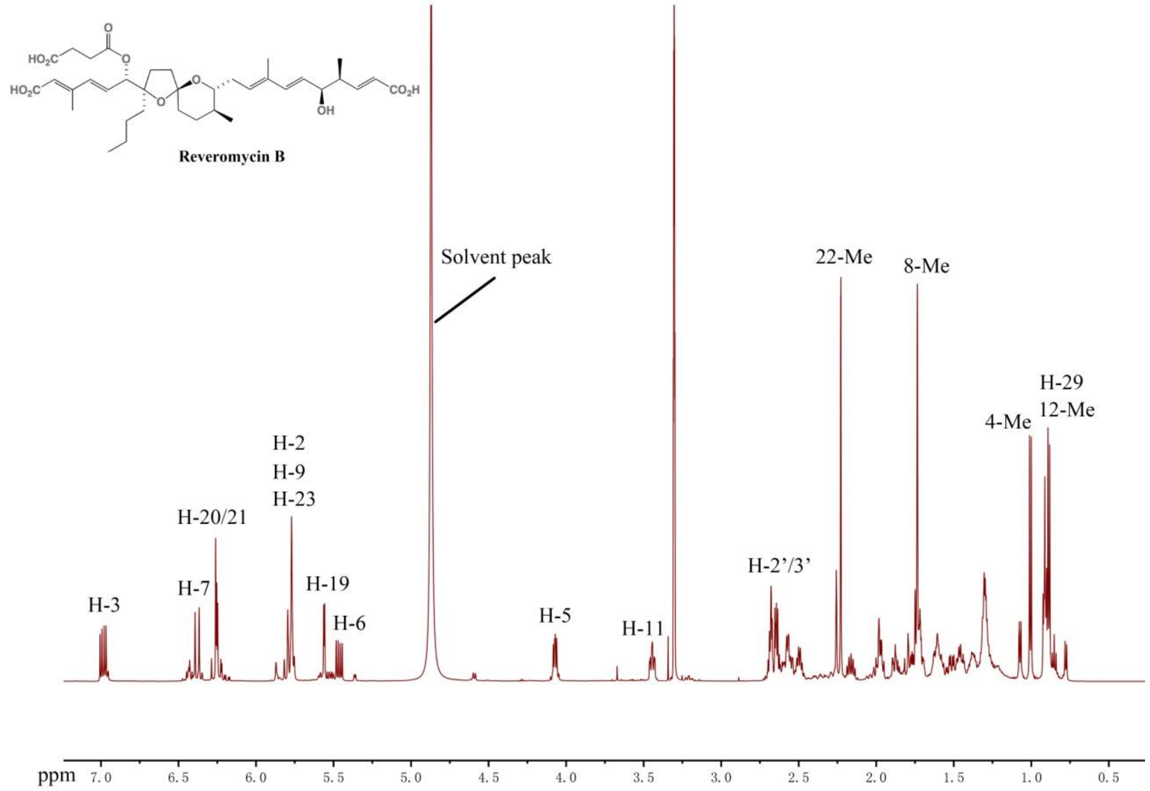

Supplementary Figure S7. ^{1}H NMR (400 MHz, MeOH-d_4, NMR) spectrum for reveromycin B from *Streptomyces* sp. 3-10.

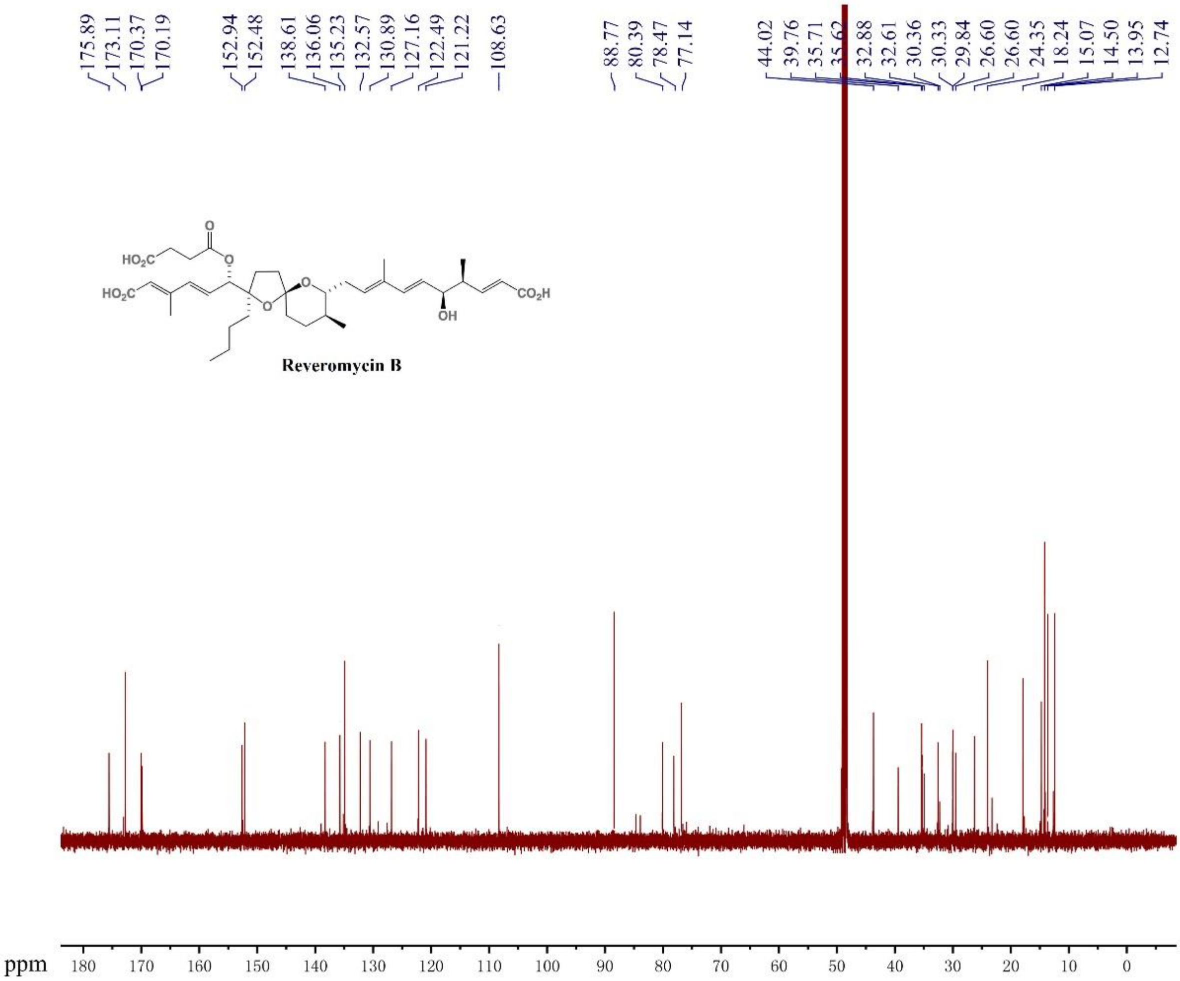

Supplementary Figure S8. ^{13}C NMR (100 MHz, MeOH-d$_4$, NMR) spectrum for reveromycin B from *Streptomyces* sp. 3-10.

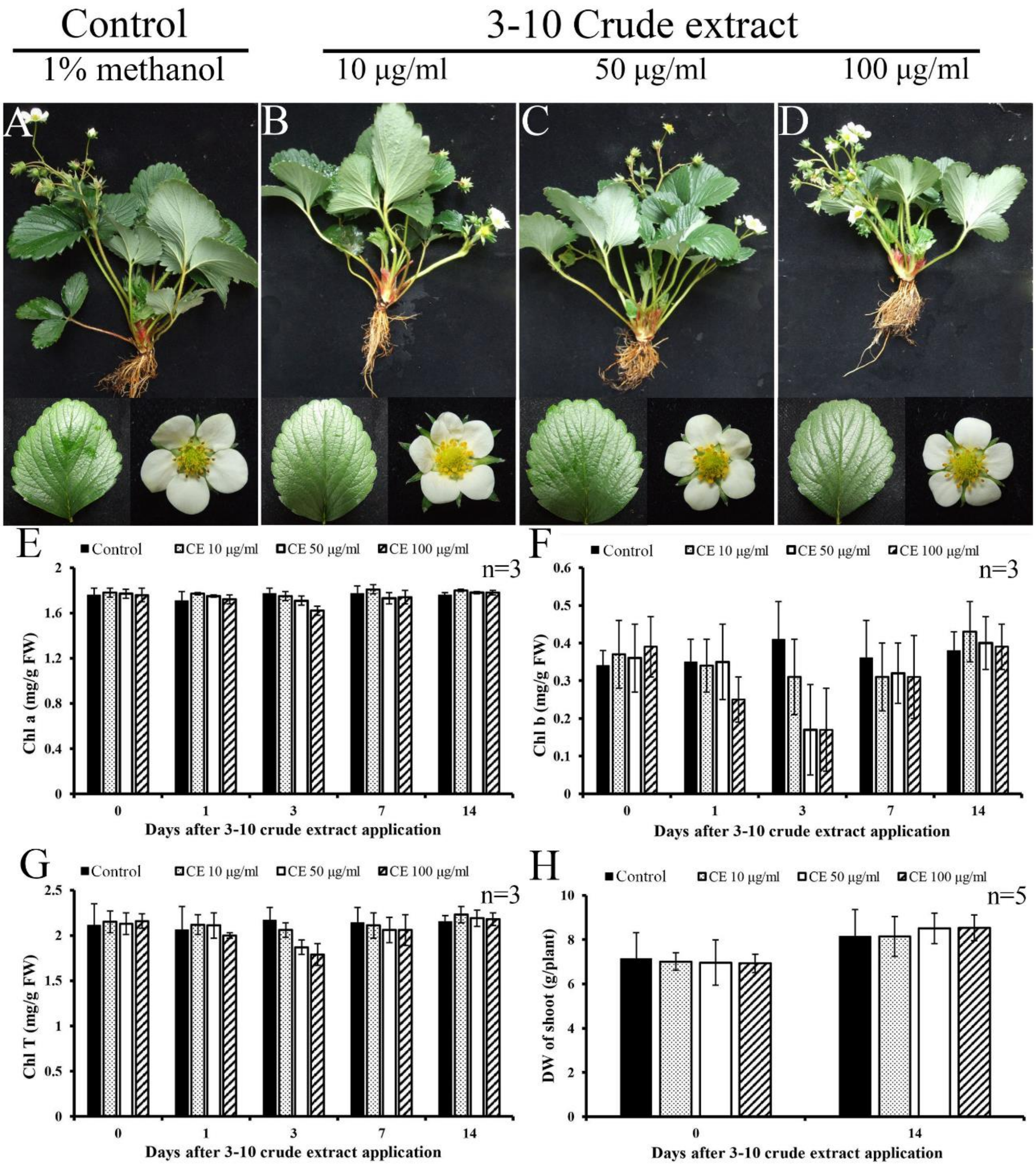

Supplementary Figure S9. Toxicity of the crude extract (CE) of *Streptomyces* sp. 3-10 to strawberry plants grown in a plastic tunnel. (**A, B, C and D)** Four strawberry plants for the treatments of control and three CE concentrations. Note the healthy appearance of the leaves and flowers; (**E, F and G)** Three histographs showing difference in content of chlorophyll a, chlorophyll b and the total chlorophyll b, respectively, among the four treatment. FW = fresh weight; (**H**) A histograph showing the difference in dry weight (DW) of the upper part of each plant among the four treatments.

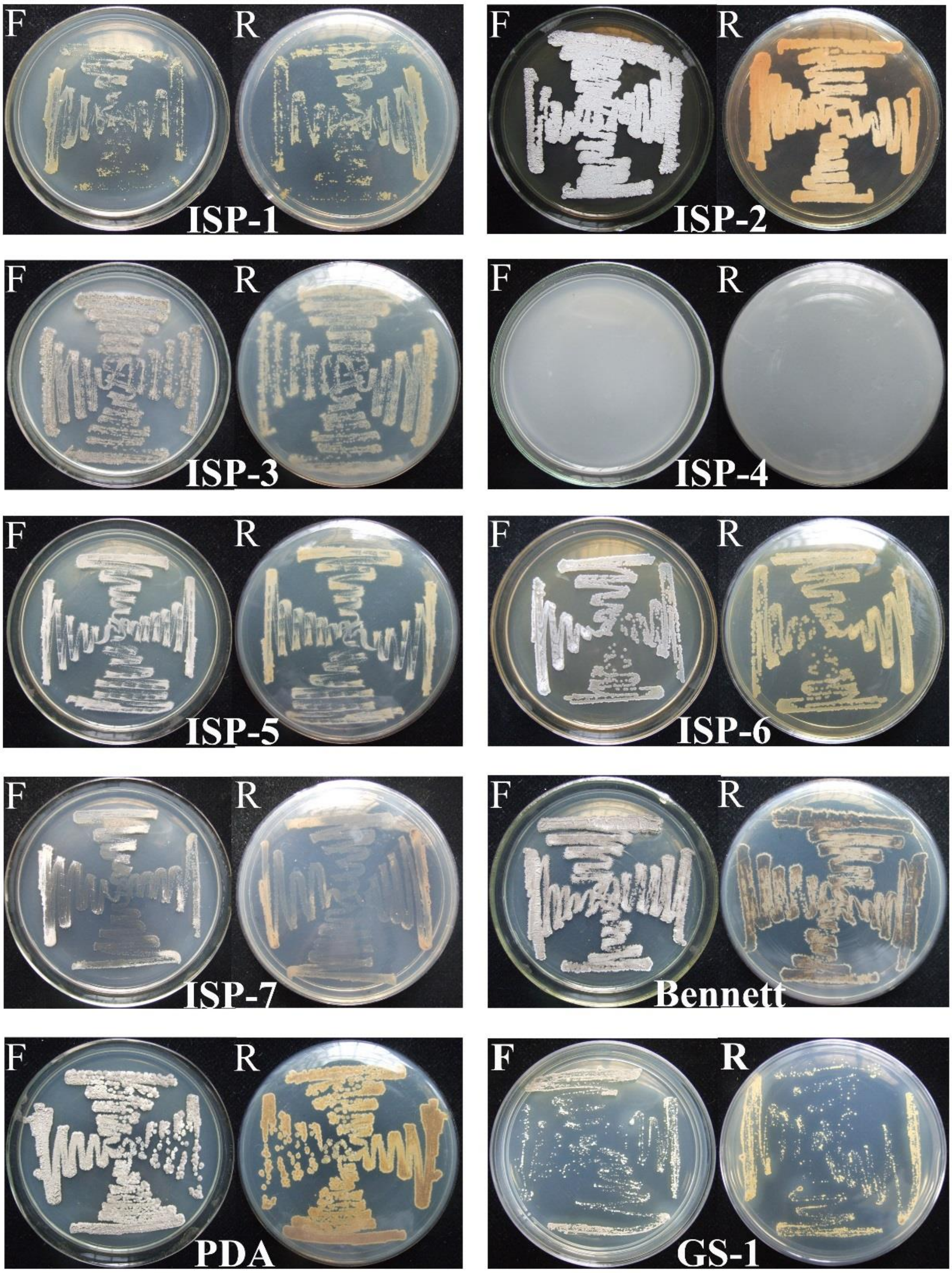

Supplementary Figure S10. Growth of *Streptomyces* sp. 3-10 on different media (28°C, 14 days, dark). **F** = front view; **R** = reversal view. ISP = International Streptomyces Project; PDA = potato dextrose agar; GS = Gauserime synthetic agar medium

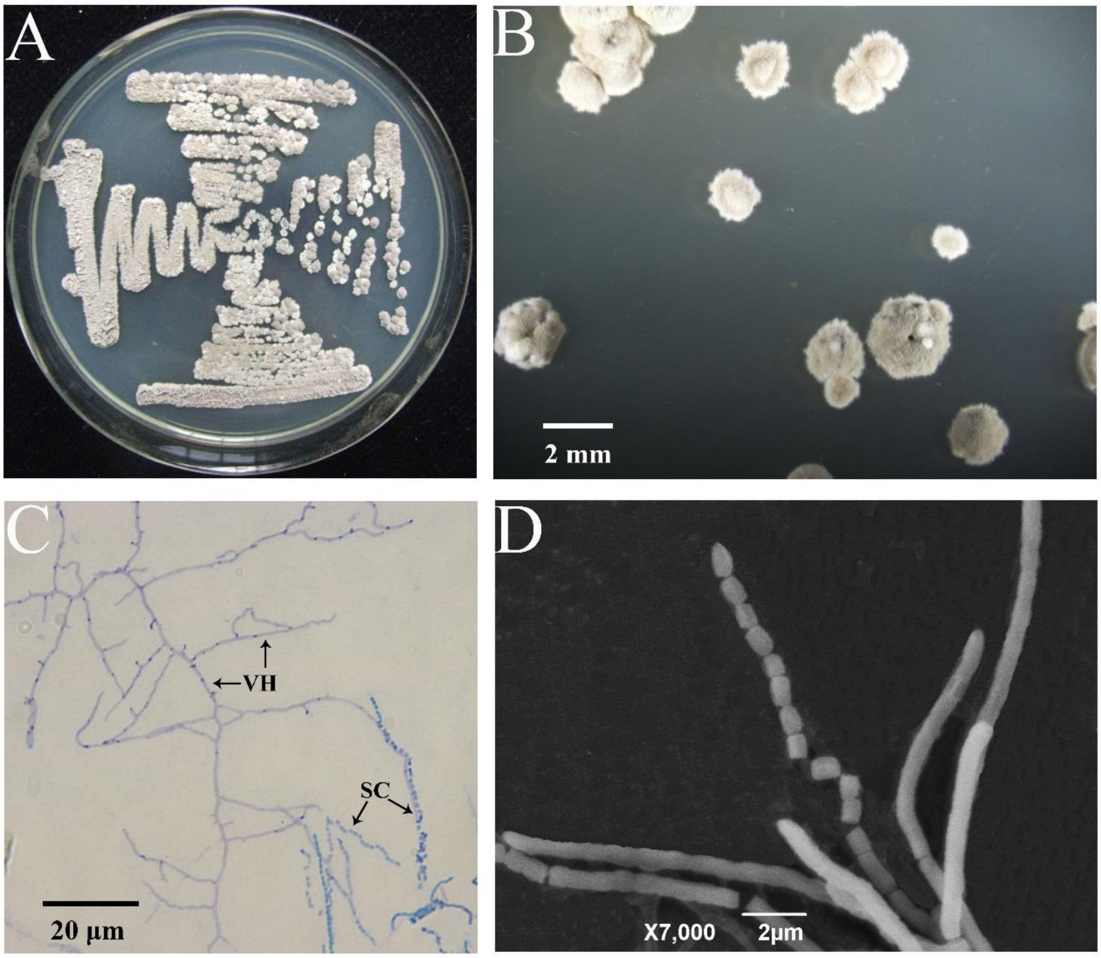

Supplementary Figure S11. Morphological features of *Streptomyces* sp. 3-10. (**A)** A 14-day-old PDA culture (28°C, dark); (**B**) Single colonies of a 14-day-old PDA culture (28°C, dark). Note the saw tooth-shaped whitish colony margin, and dome-shaped gray colony center; (**C**) Mycelia growing on a glass slide placed on a PDA culture (28°C, 14 days). They hyphae were stained with 1% methyl green. Note the non-septated vegetative hyphae (VH) and filamentous spore chain (SC); (**D**) A SEM micrograph showing the upright filamentous spore chains. Note the short rod-shaped spores.
